# Supplementary material for: Metagenomic assessment of gut microbial communities and risk of severe COVID-19
Source: Genome Med. 2023 Jul 12;15:49. doi: 10.1186/s13073-023-01202-6 (PMC10337137; doi:10.1186/s13073-023-01202-6)
Supplement: Supplementary file 1 — Additional file 1: Table S1. Participant characteristics. Table S2. Multivariable linear modeling results. Table S3. Multivariable linear modeling results. Table S4. Sensitivity analyses for differentially abundant species. Table S5. Node and network-specific information. Table S6. Multivariable linear modeling results. Figure S1. Study enrollment diagram. Figure S2. PCoA by batch. Figure S3. Volcano plot for multivariable linear modeling results. Figure S4. Entropy heatmap for clinical covariates. Figure S5. Node map. [file 13073_2023_1202_MOESM1_ESM.docx]

# Metagenomic assessment of gut microbial communities and risk of severe COVID-19

Long H. Nguyen^1,2,3^*, Daniel Okin^4^*, David A. Drew^1,2^*, Vincent M. Battista^1,2^, Sirus Jesudasen^5^, Thomas M. Kuntz^3^, Amrisha Bhosle^3,6,7^, Kelsey N. Thompson^3^, Trenton Reinicke^1,2^, Chun-Han Lo^1,2^, Jacqueline E. Woo^1,2^, Alexander Caraballo^1,2^, Lorenzo Berra^8^, Jacob Vieira^4^, Ching-Ying Huang^4^, Upasana Das Adhikari^9^, Minsik Kim^4^, Hui-Yu Sui^4^, Marina Magicheva-Gupta^1,2^, Lauren McIver^7^, Marcia B. Goldberg^10,11,12^, Douglas S. Kwon^9,10^, Curtis Huttenhower^3,6,7,11^, Andrew T. Chan^1,2,3,11^*, Peggy S. Lai^2,4^*

# Supplementary Appendix

**Table S1. Participant characteristics**

**Table S2. Multivariable linear modeling results (taxonomy)**

**Table S3. Multivariable linear modeling results (MetaCyc pathways)**

**Table S4. Sensitivity analyses for differentially abundant species**

**Table S5. Node and network-specific information**

**Table S6. Multivariable linear modeling results (predicted stool metabolites)**

**Figure S1. Study enrollment diagram**

**Figure S2. PCoA by batch**

**Figure S3. Volcano plot for multivariable linear modeling results (MetaCyc pathways)**

**Figure S4. Entropy heatmap for clinical covariates**

**Figure S5. Node map**

**Table S1. Participant characteristics.**

|  | **COVID-19 Severity** | |  |
| --- | --- | --- | --- |
|  | **Moderate** | **Severe** | ***p*-value** |
| **n** | 48 | 79 |  |
| **Age (mean (SD))** | 58.6 (12.8) | 61.0 (14.4) | 0.34 |
| **Male (%)** | 23 (47.9) | 49 (62.0) | 0.17 |
| **Race (%)** |  |  |  |
| **White** | 32 (66.7) | 48 (61.5) | 0.64 |
| **Black** | 5 (10.4) | 12 (15.4) |  |
| **Asian** | 1 (2.1) | 3 (3.8) |  |
| **American Indian** | 1 (2.1) | 0 (0.0) |  |
| **Mixed** | 1 (2.1) | 4 (5.1) |  |
| **Other** | 8 (16.7) | 11 (14.1) |  |
| **Ethnicity (%)** |  |  | 0.73 |
| **Not Hispanic** | 33 (73.3) | 50 (68.5) |  |
| **Hispanic** | 12 (26.7) | 23 (31.5) |  |
| **Body Mass Index (BMI) (mean kg/m^2^ (SD))** | 30.0 (7.7) | 33.3 (7.5) | 0.02 |
| **Comorbidities** |  |  |  |
| **Charlson Comorbidity Index (mean (SD))** | 3.0 (2.4) | 3.5 (2.4) | 0.24 |
| **Immunosuppression (%)** | 10 (20.8) | 15 (19.0) | 0.98 |
| **Cancer (%)** | 6 (12.5) | 13 (16.5) | 0.73 |
| **Pulmonary (%)** | 19 (39.6) | 30 (38.0) | 1 |
| **Cardiac (%)** | 30 (62.5) | 57 (72.2) | 0.35 |
| **Hypertension (%)** | 20 (41.7) | 47 (59.5) | 0.08 |
| **Hyperlipidemia (%)** | 16 (33.3) | 26 (32.9) | 1 |
| **Diabetes Mellitus (%)** | 10 (20.8) | 30 (38.0) | 0.07 |
| **Smoking (%)** |  |  | 0.52 |
| **Active** | 3 (6.2) | 2 (2.5) |  |
| **Former** | 17 (35.4) | 22 (27.8) |  |
| **Never** | 24 (50.0) | 46 (58.2) |  |
| **Unknown** | 4 (8.3) | 9 (11.4) |  |
| **Pack-Years among active/former (mean (SD))** | 27.7 (24.7) | 24.5 (17.1) | 0.71 |
| **Hospital Course** |  |  |  |
| **Admission SAP Score (mean (SD))** | 22.2 (9.2) | 32.0 (12.5) | <0.001 |
| **Admission SOFA Score (mean (SD))** | 2.7 (1.4) | 5.4 (2.6) | <0.001 |
| **ICU Admission (%)** | 0 (0.0) | 78 (98.7) | <0.001 |
| **Antibiotics (%)** | 14 (29.2) | 39 (49.4) | 0.04 |
| **Antiviral Therapy (%)** | 31 (64.6) | 65 (82.3) | 0.04 |
| **Remdesivir (%)** | 30 (62.5) | 48 (60.8) | 0.99 |
| **Hydroxychloroquine (%)** | 1 (2.1) | 16 (20.3) | 0.01 |
| **Corticosteroids (%)** | 33 (68.8) | 53 (67.1) | 1 |
| **Anti-IL-6 Therapy (%)** | 1 (2.1) | 7 (8.9) | 0.25 |
| **Oxygen Therapy (%)** | 39 (81.2) | 78 (98.7) | 0.001 |
| **High Flow Oxygen (%)** | 0 (0.0) | 39 (49.4) | <0.001 |
| **BPAP (%)** | 0 (0.0) | 13 (16.5) | 0.008 |
| **Mechanical Ventilation (%)** | 0 (0.0) | 74 (93.7) | <0.001 |
| **Deceased within 90 days (%)** | 2 (4.2) | 18 (22.8) | 0.01 |
| Abbreviations: BMI (body mass index), BPAP (bilevel positive airway pressure), ICU (intensive care unit), IL-6 (interleukin-6), kg (kilogram), m (meter), SAP (Simplified Acute Physiology), SD (standard deviation), SOFA (Sequential Organ Failure Assessment) | | | |

**Table S2. Multivariable linear modeling results (taxonomy)**

| **feature** | **metadata** | **value** | **coef** | **stderr** | **N** | **N.not.0** | **pval** | **qval** |
| --- | --- | --- | --- | --- | --- | --- | --- | --- |
| *Eubacterium_eligens* | covid_severity | Severe | -5.58 | 0.81 | 241 | 48 | 3.53E-10 | 5.15E-07 |
| *Eubacterium_hallii* | covid_severity | Severe | -5.02 | 0.74 | 241 | 80 | 4.05E-10 | 5.15E-07 |
| *Roseburia_hominis* | covid_severity | Severe | -5.87 | 0.87 | 241 | 48 | 7.31E-10 | 6.20E-07 |
| *Anaerostipes_hadrus* | covid_severity | Severe | -5.11 | 0.79 | 241 | 62 | 1.89E-09 | 1.20E-06 |
| *Fusicatenibacter_saccharivorans* | covid_severity | Severe | -5.66 | 0.88 | 241 | 63 | 2.97E-09 | 1.51E-06 |
| *Adlercreutzia_equolifaciens* | covid_severity | Severe | -5.04 | 0.79 | 241 | 60 | 4.39E-09 | 1.86E-06 |
| *Actinomyces_odontolyticus* | covid_severity | Severe | -2.44 | 0.38 | 241 | 52 | 5.50E-09 | 1.86E-06 |
| *Agathobaculum_butyriciproducens* | covid_severity | Severe | -3.49 | 0.56 | 241 | 55 | 5.86E-09 | 1.86E-06 |
| *Asaccharobacter_celatus* | covid_severity | Severe | -5.42 | 0.87 | 241 | 61 | 6.82E-09 | 1.93E-06 |
| *Clostridium_leptum* | covid_severity | Severe | -3.89 | 0.66 | 241 | 91 | 2.88E-08 | 7.33E-06 |
| *Roseburia_intestinalis* | covid_severity | Severe | -4.03 | 0.69 | 241 | 32 | 5.87E-08 | 1.36E-05 |
| *Blautia_wexlerae* | covid_severity | Severe | -5.00 | 0.88 | 241 | 96 | 7.77E-08 | 1.65E-05 |
| *Streptococcus_parasanguinis* | covid_severity | Severe | -4.17 | 0.73 | 241 | 78 | 8.82E-08 | 1.73E-05 |
| *Dorea_formicigenerans* | covid_severity | Severe | -4.37 | 0.80 | 241 | 56 | 2.56E-07 | 4.65E-05 |
| *Eubacterium_siraeum* | covid_severity | Severe | -4.41 | 0.81 | 241 | 45 | 2.90E-07 | 4.91E-05 |
| *Gemmiger_formicilis* | covid_severity | Severe | -3.93 | 0.72 | 241 | 49 | 3.15E-07 | 5.00E-05 |
| *Enterorhabdus_caecimuris* | covid_severity | Severe | -2.55 | 0.48 | 241 | 56 | 5.26E-07 | 7.87E-05 |
| *Eubacterium_rectale* | covid_severity | Severe | -4.37 | 0.84 | 241 | 55 | 8.59E-07 | 0.000121398 |
| *Actinomyces_sp_HMSC035G02* | covid_severity | Severe | -1.96 | 0.37 | 241 | 37 | 9.54E-07 | 0.000127756 |
| *Oscillibacter_sp_57_20* | covid_severity | Severe | -2.68 | 0.53 | 241 | 31 | 1.34E-06 | 0.000170974 |
| *Ruminococcus_bicirculans* | covid_severity | Severe | -3.53 | 0.70 | 241 | 27 | 2.02E-06 | 0.000244644 |
| *Ruminococcus_torques* | covid_severity | Severe | -3.80 | 0.77 | 241 | 57 | 2.48E-06 | 0.00028714 |
| *Streptococcus_mitis* | covid_severity | Severe | -2.41 | 0.49 | 241 | 29 | 3.21E-06 | 0.000354728 |
| *Blautia_sp_CAG_257* | covid_severity | Severe | -3.29 | 0.68 | 241 | 56 | 3.78E-06 | 0.000400776 |
| *Streptococcus_salivarius* | covid_severity | Severe | -3.18 | 0.66 | 241 | 78 | 4.69E-06 | 0.000477032 |
| *Collinsella_stercoris* | covid_severity | Severe | -2.85 | 0.60 | 241 | 69 | 5.27E-06 | 0.000496993 |
| *Rothia_mucilaginosa* | covid_severity | Severe | -2.53 | 0.53 | 241 | 41 | 5.17E-06 | 0.000496993 |
| *Roseburia_inulinivorans* | covid_severity | Severe | -3.26 | 0.68 | 241 | 46 | 5.54E-06 | 0.00050344 |
| *Streptococcus_salivarius* | time_from_admit | time_from_admit | -0.07 | 0.02 | 241 | 78 | 1.47E-05 | 0.001288644 |
| *Faecalibacterium_prausnitzii* | time_from_admit | time_from_admit | -0.11 | 0.02 | 241 | 110 | 1.59E-05 | 0.001347113 |
| *Actinomyces_sp_HPA0247* | covid_severity | Severe | -1.80 | 0.40 | 241 | 34 | 1.75E-05 | 0.001436743 |
| *Streptococcus_gordonii* | covid_severity | Severe | -1.74 | 0.40 | 241 | 34 | 2.80E-05 | 0.002229106 |
| *Streptococcus_oralis* | covid_severity | Severe | -2.06 | 0.48 | 241 | 36 | 3.45E-05 | 0.002655947 |
| *Monoglobus_pectinilyticus* | covid_severity | Severe | -2.78 | 0.66 | 241 | 31 | 4.45E-05 | 0.003327227 |
| *Firmicutes_bacterium_CAG_145* | stool_viral_avg | stool_viral_avg | 0.98 | 0.24 | 241 | 58 | 4.86E-05 | 0.003501013 |
| *Streptococcus_thermophilus* | covid_severity | Severe | -2.94 | 0.70 | 241 | 70 | 4.95E-05 | 0.003501013 |
| *Bacteroides_ovatus* | read_depth | read_depth | 1.57 | 0.38 | 241 | 153 | 5.43E-05 | 0.003736829 |
| *Actinomyces_oris* | covid_severity | Severe | -1.85 | 0.45 | 241 | 49 | 7.38E-05 | 0.004941991 |
| *Slackia_isoflavoniconvertens* | charlson | charlson | 1.32 | 0.32 | 241 | 29 | 8.36E-05 | 0.005456455 |
| *Clostridium_sp_CAG_58* | covid_severity | Severe | -2.67 | 0.67 | 241 | 36 | 9.81E-05 | 0.006237965 |
| *Dorea_longicatena* | covid_severity | Severe | -3.51 | 0.88 | 241 | 67 | 0.000101332 | 0.006287522 |
| *Bilophila_wadsworthia* | stool_viral_avg | stool_viral_avg | 0.96 | 0.25 | 241 | 83 | 0.000131041 | 0.007937357 |
| *Collinsella_aerofaciens* | covid_severity | Severe | -4.71 | 1.22 | 241 | 105 | 0.000169451 | 0.010025208 |
| *Clostridium_clostridioforme* | male | yes | -2.79 | 0.72 | 241 | 71 | 0.000180928 | 0.010228468 |
| *Coprococcus_catus* | covid_severity | Severe | -2.72 | 0.70 | 241 | 45 | 0.00017905 | 0.010228468 |
| *Eubacterium_eligens* | on_abx | TRUE | -3.00 | 0.78 | 241 | 48 | 0.000227243 | 0.012567528 |
| *Blautia_obeum* | covid_severity | Severe | -3.29 | 0.87 | 241 | 98 | 0.000246471 | 0.013340905 |
| *Roseburia_hominis* | stool_viral_avg | stool_viral_avg | 0.94 | 0.25 | 241 | 48 | 0.000263777 | 0.013980192 |
| *Eubacterium_rectale* | stool_viral_avg | stool_viral_avg | 0.82 | 0.22 | 241 | 55 | 0.000296811 | 0.01540994 |
| *Ruminococcus_gnavus* | male | yes | -3.85 | 1.03 | 241 | 101 | 0.000317686 | 0.016163838 |
| *Methanobrevibacter_smithii* | stool_viral_avg | stool_viral_avg | 1.08 | 0.30 | 241 | 81 | 0.00034851 | 0.017384503 |
| *Roseburia_faecis* | covid_severity | Severe | -2.58 | 0.70 | 241 | 36 | 0.000369361 | 0.018070286 |
| *Bifidobacterium_adolescentis* | covid_severity | Severe | -3.27 | 0.90 | 241 | 37 | 0.000405558 | 0.019286922 |
| *Christensenella_minuta* | stool_viral_avg | stool_viral_avg | 1.04 | 0.29 | 241 | 69 | 0.000409392 | 0.019286922 |
| *Prevotella_copri* | ethnicity | Hispanic | 3.91 | 1.07 | 241 | 45 | 0.000440384 | 0.020369764 |
| *Bacteroides_massiliensis* | tx_steroids | 1 | -4.04 | 1.12 | 241 | 56 | 0.000483858 | 0.021981 |
| *Eubacterium_ramulus* | covid_severity | Severe | -2.08 | 0.58 | 241 | 43 | 0.000526903 | 0.023516517 |
| *Candida_albicans* | covid_severity | Severe | 2.85 | 0.81 | 241 | 58 | 0.000559996 | 0.024562563 |
| *Ruminococcus_gnavus* | covid_severity | Severe | -4.35 | 1.24 | 241 | 101 | 0.000603029 | 0.026001779 |
| *Ruminococcus_bromii* | covid_severity | Severe | -3.75 | 1.09 | 241 | 41 | 0.000810607 | 0.034369741 |
| *Coprococcus_comes* | covid_severity | Severe | -2.88 | 0.85 | 241 | 58 | 0.000985043 | 0.041081154 |
| *Alistipes_putredinis* | stool_viral_avg | stool_viral_avg | 0.88 | 0.26 | 241 | 116 | 0.001031779 | 0.042336231 |
| *Enterorhabdus_caecimuris* | read_depth | read_depth | 0.52 | 0.16 | 241 | 56 | 0.001105246 | 0.044630891 |
| *Alistipes_finegoldii* | stool_viral_avg | stool_viral_avg | 0.86 | 0.26 | 241 | 128 | 0.001197463 | 0.047599135 |
| *Faecalicatena_orotica* | time_from_admit | time_from_admit | 0.06 | 0.02 | 241 | 33 | 0.001248359 | 0.048858837 |
| *Clostridium_symbiosum* | read_depth | read_depth | 0.97 | 0.30 | 241 | 81 | 0.001283603 | 0.049477043 |
| *Bifidobacterium_adolescentis* | on_abx | TRUE | -2.87 | 0.87 | 241 | 37 | 0.001330828 | 0.049644289 |
| *Blautia_hydrogenotrophica* | covid_severity | Severe | -2.98 | 0.91 | 241 | 79 | 0.00135305 | 0.049644289 |
| *Lactococcus_lactis* | covid_severity | Severe | -1.18 | 0.36 | 241 | 30 | 0.001351994 | 0.049644289 |
| *Slackia_isoflavoniconvertens* | age | age | -1.05 | 0.32 | 241 | 29 | 0.001365999 | 0.049644289 |
| *Bacteroides_stercoris* | read_depth | read_depth | 0.96 | 0.30 | 241 | 100 | 0.001496726 | 0.052361287 |
| *Dorea_sp_CAG_317* | covid_severity | Severe | -2.17 | 0.67 | 241 | 39 | 0.001469326 | 0.052361287 |
| *Roseburia_faecis* | on_abx | TRUE | -2.23 | 0.69 | 241 | 36 | 0.001502505 | 0.052361287 |
| *Enterococcus_faecalis* | time_from_admit | time_from_admit | 0.07 | 0.02 | 241 | 117 | 0.001537576 | 0.052859367 |
| *Staphylococcus_epidermidis* | time_from_admit | time_from_admit | 0.04 | 0.01 | 241 | 30 | 0.001564179 | 0.053056953 |
| *Enterococcus_faecium* | time_from_admit | time_from_admit | 0.08 | 0.03 | 241 | 85 | 0.001588931 | 0.053187362 |
| *Erysipelatoclostridium_ramosum* | stool_viral_avg | stool_viral_avg | 1.04 | 0.33 | 241 | 103 | 0.001711215 | 0.056536756 |
| *Bifidobacterium_longum* | covid_severity | Severe | -3.86 | 1.21 | 241 | 78 | 0.001744827 | 0.056908195 |
| *Streptococcus_salivarius* | stool_viral_avg | stool_viral_avg | 0.68 | 0.21 | 241 | 78 | 0.001777462 | 0.05723879 |
| *Eubacterium_eligens* | tx_remdesivir | 1 | 2.94 | 0.92 | 241 | 48 | 0.001864039 | 0.059276445 |
| *Clostridium_spiroforme* | covid_severity | Severe | -1.66 | 0.53 | 241 | 32 | 0.002000588 | 0.062833294 |
| *Actinomyces_sp_HPA0247* | age | age | 0.61 | 0.19 | 241 | 34 | 0.00205807 | 0.06385037 |
| *Alistipes_onderdonkii* | stool_viral_avg | stool_viral_avg | 0.25 | 0.08 | 241 | 27 | 0.002182969 | 0.066909322 |
| *Akkermansia_muciniphila* | tx_remdesivir | 1 | 4.34 | 1.39 | 241 | 65 | 0.002259041 | 0.068416668 |
| *Alistipes_putredinis* | tx_remdesivir | 1 | 3.73 | 1.20 | 241 | 116 | 0.002290425 | 0.068551062 |
| *Clostridium_scindens* | read_depth | read_depth | 0.88 | 0.29 | 241 | 82 | 0.002374711 | 0.070247255 |
| *Faecalibacterium_prausnitzii* | covid_severity | Severe | -3.22 | 1.05 | 241 | 110 | 0.002619834 | 0.076607573 |
| *Roseburia_inulinivorans* | stool_viral_avg | stool_viral_avg | 0.67 | 0.22 | 241 | 46 | 0.002781937 | 0.08042328 |
| *Anaerotruncus_colihominis* | stool_viral_avg | stool_viral_avg | 0.82 | 0.27 | 241 | 79 | 0.002854961 | 0.080575306 |
| *Bifidobacterium_adolescentis* | race | Other | -4.55 | 1.49 | 241 | 37 | 0.002913887 | 0.080575306 |
| *Collinsella_massiliensis* | covid_severity | Severe | -1.16 | 0.38 | 241 | 48 | 0.002911007 | 0.080575306 |
| *Eubacterium_hallii* | time_from_admit | time_from_admit | -0.06 | 0.02 | 241 | 80 | 0.00284829 | 0.080575306 |
| *Collinsella_aerofaciens* | stool_viral_avg | stool_viral_avg | 1.13 | 0.38 | 241 | 105 | 0.003000269 | 0.082071887 |
| *Clostridium_methylpentosum* | read_depth | read_depth | 0.53 | 0.18 | 241 | 41 | 0.003050523 | 0.082558826 |
| *Enterococcus_faecalis* | covid_severity | Severe | 2.78 | 0.93 | 241 | 117 | 0.003084942 | 0.082611489 |
| *Alistipes_putredinis* | tx_steroids | 1 | -3.75 | 1.24 | 241 | 116 | 0.003148631 | 0.083438709 |
| *Adlercreutzia_equolifaciens* | read_depth | read_depth | 0.70 | 0.23 | 241 | 60 | 0.003211461 | 0.084226354 |
| *Intestinimonas_butyriciproducens* | covid_severity | Severe | -2.58 | 0.87 | 241 | 57 | 0.003478282 | 0.090293352 |
| *Catabacter_hongkongensis* | stool_viral_avg | stool_viral_avg | 0.61 | 0.21 | 241 | 56 | 0.003551759 | 0.090643154 |
| *Streptococcus_mitis* | read_depth | read_depth | 0.50 | 0.17 | 241 | 29 | 0.003563017 | 0.090643154 |
| *Akkermansia_muciniphila* | covid_severity | Severe | -3.61 | 1.22 | 241 | 65 | 0.003657401 | 0.092123044 |
| *Lactobacillus_rhamnosus* | on_abx | TRUE | 2.79 | 0.94 | 241 | 106 | 0.00373657 | 0.093194447 |
| *Asaccharobacter_celatus* | read_depth | read_depth | 0.75 | 0.26 | 241 | 61 | 0.003790563 | 0.09362323 |
| *Pseudomonas_aeruginosa_group* | race | Black | 2.12 | 0.72 | 241 | 26 | 0.003940362 | 0.096387317 |
| *Bifidobacterium_longum* | time_from_admit | time_from_admit | -0.08 | 0.03 | 241 | 78 | 0.004181222 | 0.101305042 |
| *Intestinimonas_butyriciproducens* | stool_viral_avg | stool_viral_avg | 0.73 | 0.25 | 241 | 57 | 0.004256881 | 0.102165132 |
| *Asaccharobacter_celatus* | stool_viral_avg | stool_viral_avg | 0.68 | 0.23 | 241 | 61 | 0.004402636 | 0.104675752 |
| *Bifidobacterium_adolescentis* | male | yes | -2.21 | 0.76 | 241 | 37 | 0.004671986 | 0.106458453 |
| *Coprococcus_catus* | age | age | -1.04 | 0.36 | 241 | 45 | 0.004545777 | 0.106458453 |
| *Coprococcus_comes* | age | age | -1.24 | 0.43 | 241 | 58 | 0.00468685 | 0.106458453 |
| *Parabacteroides_distasonis* | tx_remdesivir | 1 | 3.91 | 1.36 | 241 | 164 | 0.004628298 | 0.106458453 |
| *Roseburia_inulinivorans* | time_from_admit | time_from_admit | -0.05 | 0.02 | 241 | 46 | 0.004661124 | 0.106458453 |
| *Bifidobacterium_longum* | stool_viral_avg | stool_viral_avg | 1.03 | 0.36 | 241 | 78 | 0.005014341 | 0.112043291 |
| *Butyricimonas_synergistica* | age | age | -0.91 | 0.32 | 241 | 37 | 0.005020808 | 0.112043291 |
| *Clostridium_sp_CAG_58* | age | age | -0.92 | 0.32 | 241 | 36 | 0.00525945 | 0.116348175 |
| *Gordonibacter_pamelaeae* | stool_viral_avg | stool_viral_avg | 0.73 | 0.26 | 241 | 164 | 0.005320728 | 0.116689063 |
| *Firmicutes_bacterium_CAG_145* | covid_severity | Severe | -2.48 | 0.88 | 241 | 58 | 0.005381672 | 0.117016866 |
| *Parabacteroides_merdae* | stool_viral_avg | stool_viral_avg | 0.80 | 0.28 | 241 | 126 | 0.005428295 | 0.117030351 |
| *Clostridium_bolteae* | time_from_admit | time_from_admit | -0.06 | 0.02 | 241 | 112 | 0.005583849 | 0.119372375 |
| *Bilophila_wadsworthia* | covid_severity | Severe | -2.33 | 0.83 | 241 | 83 | 0.005738971 | 0.120660689 |
| *Eisenbergiella_tayi* | covid_severity | Severe | -2.72 | 0.97 | 241 | 92 | 0.005708216 | 0.120660689 |
| *Streptococcus_gordonii* | race | American Indian | 5.53 | 1.97 | 241 | 34 | 0.005813717 | 0.121086244 |
| *Streptococcus_oralis* | race | Black | 1.59 | 0.55 | 241 | 36 | 0.005854406 | 0.121086244 |
| *Lactobacillus_rhamnosus* | time_from_admit | time_from_admit | 0.07 | 0.02 | 241 | 106 | 0.006013572 | 0.123375225 |
| *Ruminococcus_bromii* | stool_viral_avg | stool_viral_avg | 0.86 | 0.31 | 241 | 41 | 0.006079994 | 0.123740038 |
| *Alistipes_shahii* | stool_viral_avg | stool_viral_avg | 0.96 | 0.35 | 241 | 106 | 0.00614932 | 0.124157699 |
| *Coprococcus_catus* | on_abx | TRUE | -1.90 | 0.68 | 241 | 45 | 0.006299679 | 0.126191996 |
| *Clostridium_innocuum* | read_depth | read_depth | 1.19 | 0.43 | 241 | 168 | 0.006559481 | 0.129359065 |
| *Dorea_formicigenerans* | time_from_admit | time_from_admit | -0.05 | 0.02 | 241 | 56 | 0.006517488 | 0.129359065 |
| *Bacteroides_vulgatus* | time_from_admit | time_from_admit | -0.06 | 0.02 | 241 | 171 | 0.006642948 | 0.129997386 |
| *Adlercreutzia_equolifaciens* | stool_viral_avg | stool_viral_avg | 0.59 | 0.21 | 241 | 60 | 0.006702226 | 0.130156207 |
| *Clostridium_citroniae* | read_depth | read_depth | 0.81 | 0.30 | 241 | 66 | 0.00709512 | 0.135708076 |
| *Enterococcus_avium* | ethnicity | Hispanic | 2.04 | 0.74 | 241 | 28 | 0.007069445 | 0.135708076 |
| *Lawsonibacter_asaccharolyticus* | read_depth | read_depth | 0.84 | 0.31 | 241 | 103 | 0.007148145 | 0.135708076 |
| *Dorea_formicigenerans* | race | Mixed | -4.88 | 1.78 | 241 | 56 | 0.007255365 | 0.136723332 |
| *Clostridium_scindens* | ethnicity | Hispanic | -2.53 | 0.92 | 241 | 82 | 0.007459094 | 0.139528941 |
| *Klebsiella_variicola* | time_from_admit | time_from_admit | 0.04 | 0.02 | 241 | 26 | 0.007597662 | 0.141083591 |
| *Clostridium_sp_CAG_58* | tx_remdesivir | 1 | 2.03 | 0.75 | 241 | 36 | 0.007672947 | 0.141449106 |
| *Oscillibacter_sp_57_20* | race | Other | 2.41 | 0.89 | 241 | 31 | 0.007729655 | 0.141469363 |
| *Blautia_wexlerae* | time_from_admit | time_from_admit | -0.06 | 0.02 | 241 | 96 | 0.007808002 | 0.141882557 |
| *Agathobaculum_butyriciproducens* | on_abx | TRUE | -1.46 | 0.54 | 241 | 55 | 0.0079358 | 0.1431821 |
| *Butyricimonas_synergistica* | race | American Indian | 8.16 | 3.02 | 241 | 37 | 0.008049207 | 0.143418374 |
| *Rothia_mucilaginosa* | read_depth | read_depth | 0.43 | 0.16 | 241 | 41 | 0.008061646 | 0.143418374 |
| *Bacteroides_intestinalis* | race | American Indian | 8.51 | 3.17 | 241 | 26 | 0.008365406 | 0.147110089 |
| *Fusicatenibacter_saccharivorans* | time_from_admit | time_from_admit | -0.05 | 0.02 | 241 | 63 | 0.008384812 | 0.147110089 |
| *Collinsella_stercoris* | stool_viral_avg | stool_viral_avg | 0.47 | 0.18 | 241 | 69 | 0.008452141 | 0.147275661 |
| *Sellimonas_intestinalis* | stool_viral_avg | stool_viral_avg | 0.84 | 0.32 | 241 | 101 | 0.008674535 | 0.150122563 |
| *Bifidobacterium_adolescentis* | age | age | -1.21 | 0.45 | 241 | 37 | 0.008980572 | 0.154137839 |
| *Streptococcus_vestibularis* | race | American Indian | 7.98 | 3.01 | 241 | 47 | 0.009027727 | 0.154137839 |
| *Eubacterium_rectale* | time_from_admit | time_from_admit | -0.04 | 0.02 | 241 | 55 | 0.009242868 | 0.155720901 |
| *Methanobrevibacter_smithii* | covid_severity | Severe | -2.93 | 1.11 | 241 | 81 | 0.009187788 | 0.155720901 |
| *Rothia_dentocariosa* | race | American Indian | 4.96 | 1.88 | 241 | 28 | 0.009328019 | 0.156121577 |
| *Anaerofustis_stercorihominis* | stool_viral_avg | stool_viral_avg | 0.46 | 0.18 | 241 | 44 | 0.009495802 | 0.157837168 |
| *Staphylococcus_epidermidis* | read_depth | read_depth | -0.53 | 0.20 | 241 | 30 | 0.009554608 | 0.157837168 |
| *Clostridium_leptum* | stool_viral_avg | stool_viral_avg | 0.51 | 0.19 | 241 | 91 | 0.009646923 | 0.158334007 |
| *Bifidobacterium_adolescentis* | time_from_admit | time_from_admit | -0.05 | 0.02 | 241 | 37 | 0.010534492 | 0.170705317 |
| *Parabacteroides_johnsonii* | race | American Indian | 6.91 | 2.66 | 241 | 25 | 0.01053488 | 0.170705317 |
| *Dorea_formicigenerans* | ethnicity | Hispanic | 2.53 | 0.97 | 241 | 56 | 0.010847738 | 0.173563814 |
| *Streptococcus_vestibularis* | stool_viral_avg | stool_viral_avg | 0.52 | 0.20 | 241 | 47 | 0.010813357 | 0.173563814 |
| *Intestinimonas_butyriciproducens* | time_from_admit | time_from_admit | -0.05 | 0.02 | 241 | 57 | 0.011242704 | 0.17875899 |
| *Bacteroides_cellulosilyticus* | time_from_admit | time_from_admit | -0.05 | 0.02 | 241 | 51 | 0.011637323 | 0.183884159 |
| *Anaeromassilibacillus_sp_An250* | charlson | charlson | -0.64 | 0.25 | 241 | 31 | 0.011711613 | 0.183915696 |
| *Acidaminococcus_intestini* | read_depth | read_depth | 0.79 | 0.31 | 241 | 43 | 0.011846146 | 0.184887094 |
| *Agathobaculum_butyriciproducens* | ethnicity | Hispanic | 1.73 | 0.68 | 241 | 55 | 0.012300713 | 0.188160045 |
| *Clostridium_clostridioforme* | tx_remdesivir | 1 | 2.57 | 1.01 | 241 | 71 | 0.012273133 | 0.188160045 |
| *Gordonibacter_pamelaeae* | time_from_admit | time_from_admit | -0.05 | 0.02 | 241 | 164 | 0.012351701 | 0.188160045 |
| *Streptococcus_vestibularis* | time_from_admit | time_from_admit | -0.04 | 0.01 | 241 | 47 | 0.012254755 | 0.188160045 |
| *Akkermansia_muciniphila* | stool_viral_avg | stool_viral_avg | 0.87 | 0.35 | 241 | 65 | 0.012468531 | 0.188590585 |
| *Anaerotruncus_colihominis* | charlson | charlson | 1.02 | 0.40 | 241 | 79 | 0.012750621 | 0.188590585 |
| *Coprococcus_catus* | time_from_admit | time_from_admit | -0.04 | 0.01 | 241 | 45 | 0.012713554 | 0.188590585 |
| *Klebsiella_pneumoniae* | time_from_admit | time_from_admit | 0.04 | 0.01 | 241 | 31 | 0.012643431 | 0.188590585 |
| *Roseburia_inulinivorans* | race | Mixed | -3.80 | 1.49 | 241 | 46 | 0.012665439 | 0.188590585 |
| *Ruthenibacterium_lactatiformans* | read_depth | read_depth | 1.02 | 0.41 | 241 | 183 | 0.013287639 | 0.195397414 |
| *Faecalicatena_orotica* | read_depth | read_depth | 0.66 | 0.27 | 241 | 33 | 0.013598293 | 0.198816425 |
| *Collinsella_aerofaciens* | time_from_admit | time_from_admit | -0.07 | 0.03 | 241 | 105 | 0.013756706 | 0.198846663 |
| *Holdemanella_biformis* | covid_severity | Severe | -1.51 | 0.61 | 241 | 42 | 0.013808989 | 0.198846663 |
| *Staphylococcus_epidermidis* | on_abx | TRUE | -1.06 | 0.42 | 241 | 30 | 0.01383485 | 0.198846663 |
| *Staphylococcus_epidermidis* | race | Black | -1.37 | 0.55 | 241 | 30 | 0.014209952 | 0.202348345 |
| *Streptococcus_parasanguinis* | time_from_admit | time_from_admit | -0.05 | 0.02 | 241 | 78 | 0.01423756 | 0.202348345 |
| *Anaerostipes_hadrus* | time_from_admit | time_from_admit | -0.04 | 0.02 | 241 | 62 | 0.014463476 | 0.204417126 |
| *Rothia_dentocariosa* | covid_severity | Severe | -0.94 | 0.38 | 241 | 28 | 0.014715218 | 0.206826041 |
| *Eisenbergiella_massiliensis* | stool_viral_avg | stool_viral_avg | 0.55 | 0.22 | 241 | 63 | 0.015029637 | 0.20868462 |
| *Eubacterium_eligens* | male | yes | 1.68 | 0.68 | 241 | 48 | 0.015093542 | 0.20868462 |
| *Roseburia_inulinivorans* | on_abx | TRUE | -1.61 | 0.65 | 241 | 46 | 0.01497856 | 0.20868462 |
| *Eubacterium_siraeum* | male | yes | 1.74 | 0.71 | 241 | 45 | 0.015939887 | 0.219194993 |
| *Clostridium_sp_CAG_58* | race | Asian | 3.47 | 1.41 | 241 | 36 | 0.016190282 | 0.221441277 |
| *Blautia_coccoides* | read_depth | read_depth | 0.83 | 0.34 | 241 | 84 | 0.016821983 | 0.225237495 |
| *Gemmiger_formicilis* | age | age | -0.88 | 0.36 | 241 | 49 | 0.016651376 | 0.225237495 |
| *Lawsonibacter_asaccharolyticus* | tx_remdesivir | 1 | 2.33 | 0.96 | 241 | 103 | 0.01680232 | 0.225237495 |
| *Veillonella_parvula* | read_depth | read_depth | 0.78 | 0.32 | 241 | 45 | 0.016787589 | 0.225237495 |
| *Anaerofustis_stercorihominis* | charlson | charlson | 0.71 | 0.29 | 241 | 44 | 0.017119364 | 0.226831579 |
| *Proteobacteria_bacterium_CAG_139* | male | yes | -1.40 | 0.58 | 241 | 57 | 0.017073037 | 0.226831579 |
| *Eubacterium_rectale* | on_abx | TRUE | -1.98 | 0.82 | 241 | 55 | 0.017228755 | 0.227098203 |
| *Clostridium_scindens* | stool_viral_avg | stool_viral_avg | 0.62 | 0.26 | 241 | 82 | 0.017741012 | 0.232645029 |
| *Fusicatenibacter_saccharivorans* | on_abx | TRUE | -2.05 | 0.85 | 241 | 63 | 0.018176371 | 0.237131729 |
| *Odoribacter_splanchnicus* | on_abx | TRUE | -2.18 | 0.91 | 241 | 107 | 0.018525277 | 0.239229977 |
| *Slackia_isoflavoniconvertens* | male | yes | 1.29 | 0.54 | 241 | 29 | 0.018484946 | 0.239229977 |
| *Bifidobacterium_breve* | age | age | -0.92 | 0.38 | 241 | 28 | 0.018953186 | 0.239885098 |
| *Candida_albicans* | read_depth | read_depth | -0.72 | 0.30 | 241 | 58 | 0.018820619 | 0.239885098 |
| *Streptococcus_anginosus_group* | race | American Indian | 8.35 | 3.52 | 241 | 58 | 0.018932468 | 0.239885098 |
| *Veillonella_parvula* | race | American Indian | 10.14 | 4.27 | 241 | 45 | 0.018891172 | 0.239885098 |
| *Bacteroides_cellulosilyticus* | stool_viral_avg | stool_viral_avg | 0.65 | 0.27 | 241 | 51 | 0.019445057 | 0.243685843 |
| *Lactobacillus_reuteri* | covid_severity | Severe | -1.38 | 0.58 | 241 | 27 | 0.019358656 | 0.243685843 |
| *Faecalibacterium_prausnitzii* | on_abx | TRUE | -2.39 | 1.01 | 241 | 110 | 0.019723042 | 0.245957935 |
| *Erysipelatoclostridium_ramosum* | covid_severity | Severe | -2.46 | 1.05 | 241 | 103 | 0.020138427 | 0.249661684 |
| *Turicimonas_muris* | male | yes | -0.99 | 0.42 | 241 | 49 | 0.020216316 | 0.249661684 |
| Results with FDR-corrected p-value <0.25 shown. | | | | | | | | |

**Table S3. Multivariable linear modeling results (MetaCyc pathways)**

| **feature** | **metadata** | **value** | **coef** | **stderr** | **N** | **N.not.0** | **pval** | **qval** |
| --- | --- | --- | --- | --- | --- | --- | --- | --- |
| PWY.7046..4.coumarate.degradation..anaerobic. | covid_severity | Severe | -2.69 | 0.45 | 241 | 101 | 1.86E-08 | 0.000120124 |
| PWY.7228..superpathway.of.guanosine.nucleotides.de.novo.biosynthesis.I | covid_severity | Severe | 0.71 | 0.14 | 241 | 241 | 1.59E-06 | 0.005123916 |
| PWY.7663..gondoate.biosynthesis..anaerobic. | covid_severity | Severe | 0.77 | 0.16 | 241 | 241 | 2.60E-06 | 0.005612303 |
| FERMENTATION.PWY..mixed.acid.fermentation | covid_severity | Severe | -2.42 | 0.53 | 241 | 194 | 9.69E-06 | 0.015668685 |
| METH.ACETATE.PWY..methanogenesis.from.acetate | covid_severity | Severe | -2.49 | 0.59 | 241 | 146 | 4.18E-05 | 0.0277281 |
| PWY.5177..glutaryl.CoA.degradation | covid_severity | Severe | -2.22 | 0.53 | 241 | 200 | 4.29E-05 | 0.0277281 |
| PWY.5973..cis.vaccenate.biosynthesis | covid_severity | Severe | 0.67 | 0.15 | 241 | 241 | 2.27E-05 | 0.0277281 |
| PWY.6895..superpathway.of.thiamin.diphosphate.biosynthesis.II | covid_severity | Severe | -2.54 | 0.59 | 241 | 146 | 3.23E-05 | 0.0277281 |
| PWY.7209..superpathway.of.pyrimidine.ribonucleosides.degradation | covid_severity | Severe | -2.32 | 0.53 | 241 | 90 | 2.61E-05 | 0.0277281 |
| PYRIDNUCSAL.PWY..NAD.salvage.pathway.I | covid_severity | Severe | -2.47 | 0.59 | 241 | 172 | 4.15E-05 | 0.0277281 |
| MET.SAM.PWY..superpathway.of.S.adenosyl.L.methionine.biosynthesis | covid_severity | Severe | -1.72 | 0.43 | 241 | 226 | 7.81E-05 | 0.031198869 |
| METSYN.PWY..L.homoserine.and.L.methionine.biosynthesis | covid_severity | Severe | -1.64 | 0.41 | 241 | 226 | 9.02E-05 | 0.031198869 |
| PPGPPMET.PWY..ppGpp.biosynthesis | covid_severity | Severe | -2.74 | 0.67 | 241 | 192 | 7.20E-05 | 0.031198869 |
| PWY.5188..tetrapyrrole.biosynthesis.I..from.glutamate. | covid_severity | Severe | -1.93 | 0.48 | 241 | 226 | 9.61E-05 | 0.031198869 |
| PWY.6125..superpathway.of.guanosine.nucleotides.de.novo.biosynthesis.II | covid_severity | Severe | 0.59 | 0.14 | 241 | 240 | 7.28E-05 | 0.031198869 |
| PWY.6891..thiazole.biosynthesis.II..Bacillus. | covid_severity | Severe | -2.64 | 0.64 | 241 | 146 | 6.69E-05 | 0.031198869 |
| PWY.7003..glycerol.degradation.to.butanol | covid_severity | Severe | -1.91 | 0.48 | 241 | 183 | 9.65E-05 | 0.031198869 |
| PWY.7383..anaerobic.energy.metabolism..invertebrates..cytosol. | covid_severity | Severe | -1.98 | 0.48 | 241 | 201 | 6.47E-05 | 0.031198869 |
| PWY.7392..taxadiene.biosynthesis..engineered. | covid_severity | Severe | -2.76 | 0.68 | 241 | 98 | 8.72E-05 | 0.031198869 |
| PWY66.399..gluconeogenesis.III | covid_severity | Severe | -1.79 | 0.45 | 241 | 199 | 8.81E-05 | 0.031198869 |
| GLYCOGENSYNTH.PWY..glycogen.biosynthesis.I..from.ADP.D.Glucose. | covid_severity | Severe | -1.33 | 0.34 | 241 | 236 | 0.000137264 | 0.034351109 |
| HOMOSER.METSYN.PWY..L.methionine.biosynthesis.I | covid_severity | Severe | -1.72 | 0.44 | 241 | 226 | 0.000124356 | 0.034351109 |
| PWY.5028..L.histidine.degradation.II | time_from_admit | time_from_admit | 0.05 | 0.01 | 241 | 66 | 0.000144811 | 0.034351109 |
| PWY.5347..superpathway.of.L.methionine.biosynthesis..transsulfuration. | covid_severity | Severe | -1.55 | 0.40 | 241 | 226 | 0.000148775 | 0.034351109 |
| PWY.5505..L.glutamate.and.L.glutamine.biosynthesis | covid_severity | Severe | -2.42 | 0.61 | 241 | 177 | 0.000116027 | 0.034351109 |
| PWY.5676..acetyl.CoA.fermentation.to.butanoate.II | covid_severity | Severe | -2.01 | 0.51 | 241 | 202 | 0.000135167 | 0.034351109 |
| PWY.6396..superpathway.of.2.3.butanediol.biosynthesis | charlson | charlson | 0.68 | 0.17 | 241 | 53 | 0.000139923 | 0.034351109 |
| PWY.7115..C4.photosynthetic.carbon.assimilation.cycle..NAD.ME.type | covid_severity | Severe | -2.48 | 0.63 | 241 | 183 | 0.000129138 | 0.034351109 |
| PWY.7456..mannan.degradation | read_depth | read_depth | 0.81 | 0.21 | 241 | 201 | 0.000172108 | 0.038368121 |
| METH.ACETATE.PWY..methanogenesis.from.acetate | time_from_admit | time_from_admit | -0.05 | 0.01 | 241 | 146 | 0.000239688 | 0.04070988 |
| PWY.241..C4.photosynthetic.carbon.assimilation.cycle..NADP.ME.type | covid_severity | Severe | -1.92 | 0.51 | 241 | 214 | 0.000245582 | 0.04070988 |
| PWY.5121..superpathway.of.geranylgeranyl.diphosphate.biosynthesis.II..via.MEP. | covid_severity | Severe | -2.55 | 0.67 | 241 | 101 | 0.000230457 | 0.04070988 |
| PWY.6353..purine.nucleotides.degradation.II..aerobic. | covid_severity | Severe | -1.94 | 0.52 | 241 | 202 | 0.000235579 | 0.04070988 |
| PWY.6549..L.glutamine.biosynthesis.III | covid_severity | Severe | -1.81 | 0.48 | 241 | 200 | 0.00024263 | 0.04070988 |
| PWY.6595..superpathway.of.guanosine.nucleotides.degradation..plants. | read_depth | read_depth | 0.73 | 0.19 | 241 | 209 | 0.000207692 | 0.04070988 |
| PWY.7220..adenosine.deoxyribonucleotides.de.novo.biosynthesis.II | covid_severity | Severe | 0.43 | 0.11 | 241 | 241 | 0.000242783 | 0.04070988 |
| PWY.7222..guanosine.deoxyribonucleotides.de.novo.biosynthesis.II | covid_severity | Severe | 0.43 | 0.11 | 241 | 241 | 0.000242783 | 0.04070988 |
| PWY66.388..fatty.acid..alpha..oxidation.III | race | Black | 1.99 | 0.51 | 241 | 43 | 0.000211707 | 0.04070988 |
| SALVADEHYPOX.PWY..adenosine.nucleotides.degradation.II | covid_severity | Severe | -2.10 | 0.55 | 241 | 203 | 0.000200181 | 0.04070988 |
| PWY.7332..superpathway.of.UDP.N.acetylglucosamine.derived.O.antigen.building.blocks.biosynthesis | read_depth | read_depth | 0.83 | 0.22 | 241 | 160 | 0.000253427 | 0.040960158 |
| PWY.5181..toluene.degradation.III..aerobic...via.p.cresol. | time_from_admit | time_from_admit | 0.04 | 0.01 | 241 | 43 | 0.000333688 | 0.051364185 |
| PWY0.781..aspartate.superpathway | covid_severity | Severe | -2.30 | 0.63 | 241 | 160 | 0.000332312 | 0.051364185 |
| P4.PWY..superpathway.of.L.lysine..L.threonine.and.L.methionine.biosynthesis.I | covid_severity | Severe | -2.33 | 0.64 | 241 | 161 | 0.000344594 | 0.051795018 |
| PWY.5941..glycogen.degradation.II..eukaryotic. | read_depth | read_depth | 0.91 | 0.25 | 241 | 189 | 0.000352511 | 0.051795018 |
| PWY.6588..pyruvate.fermentation.to.acetone | covid_severity | Severe | -2.02 | 0.57 | 241 | 190 | 0.000445671 | 0.064028094 |
| PWY66.409..superpathway.of.purine.nucleotide.salvage | read_depth | read_depth | 0.53 | 0.15 | 241 | 227 | 0.000456243 | 0.064121928 |
| PWY.4984..urea.cycle | read_depth | read_depth | 0.77 | 0.22 | 241 | 225 | 0.000481855 | 0.065100834 |
| PWY.7560..methylerythritol.phosphate.pathway.II | covid_severity | Severe | -2.48 | 0.69 | 241 | 102 | 0.000483347 | 0.065100834 |
| GOLPDLCAT.PWY..superpathway.of.glycerol.degradation.to.1.3.propanediol | covid_severity | Severe | -2.10 | 0.59 | 241 | 205 | 0.000510221 | 0.067317975 |
| PWY0.1297..superpathway.of.purine.deoxyribonucleosides.degradation | time_from_admit | time_from_admit | 0.04 | 0.01 | 241 | 231 | 0.000561158 | 0.072557791 |
| NONMEVIPP.PWY..methylerythritol.phosphate.pathway.I | covid_severity | Severe | -2.72 | 0.77 | 241 | 102 | 0.000615629 | 0.076567719 |
| PWY.6270..isoprene.biosynthesis.I | covid_severity | Severe | -2.86 | 0.82 | 241 | 102 | 0.000615858 | 0.076567719 |
| ARGSYNBSUB.PWY..L.arginine.biosynthesis.II..acetyl.cycle. | covid_severity | Severe | -1.29 | 0.37 | 241 | 237 | 0.000698707 | 0.084102993 |
| PWY.7196..superpathway.of.pyrimidine.ribonucleosides.salvage | covid_severity | Severe | -1.50 | 0.44 | 241 | 205 | 0.000702484 | 0.084102993 |
| P161.PWY..acetylene.degradation | time_from_admit | time_from_admit | 0.05 | 0.01 | 241 | 217 | 0.000734155 | 0.086296632 |
| PWY.7385..1.3.propanediol.biosynthesis..engineered. | time_from_admit | time_from_admit | -0.05 | 0.01 | 241 | 80 | 0.000748387 | 0.086398651 |
| P621.PWY..nylon.6.oligomer.degradation | read_depth | read_depth | 0.92 | 0.27 | 241 | 134 | 0.000774614 | 0.087415743 |
| PWY.7117..C4.photosynthetic.carbon.assimilation.cycle..PEPCK.type | covid_severity | Severe | -1.90 | 0.56 | 241 | 214 | 0.000795859 | 0.087415743 |
| PWY.7312..dTDP.D..beta..fucofuranose.biosynthesis | read_depth | read_depth | 0.66 | 0.19 | 241 | 91 | 0.000797762 | 0.087415743 |
| P461.PWY..hexitol.fermentation.to.lactate..formate..ethanol.and.acetate | time_from_admit | time_from_admit | 0.04 | 0.01 | 241 | 229 | 0.000828136 | 0.089231706 |
| P162.PWY..L.glutamate.degradation.V..via.hydroxyglutarate. | read_depth | read_depth | 0.68 | 0.20 | 241 | 98 | 0.000883652 | 0.093652656 |
| DAPLYSINESYN.PWY..L.lysine.biosynthesis.I | covid_severity | Severe | -2.35 | 0.69 | 241 | 198 | 0.000922557 | 0.096198936 |
| PWY.5913..TCA.cycle.VI..obligate.autotrophs. | covid_severity | Severe | -1.96 | 0.59 | 241 | 214 | 0.001068826 | 0.106384584 |
| PWY.7560..methylerythritol.phosphate.pathway.II | time_from_admit | time_from_admit | -0.05 | 0.02 | 241 | 102 | 0.001059311 | 0.106384584 |
| PWY0.1479..tRNA.processing | covid_severity | Severe | -1.93 | 0.58 | 241 | 184 | 0.001069605 | 0.106384584 |
| PWY.5265..peptidoglycan.biosynthesis.II..staphylococci. | covid_severity | Severe | 1.69 | 0.51 | 241 | 187 | 0.001117203 | 0.106455167 |
| PWY.6969..TCA.cycle.V..2.oxoglutarate.ferredoxin.oxidoreductase. | covid_severity | Severe | -1.88 | 0.57 | 241 | 191 | 0.001119714 | 0.106455167 |
| PWY66.389..phytol.degradation | charlson | charlson | 1.26 | 0.37 | 241 | 178 | 0.001116644 | 0.106455167 |
| PWY.5030..L.histidine.degradation.III | time_from_admit | time_from_admit | -0.05 | 0.02 | 241 | 216 | 0.001207111 | 0.112088394 |
| PWY.6185..4.methylcatechol.degradation..ortho.cleavage. | time_from_admit | time_from_admit | 0.04 | 0.01 | 241 | 45 | 0.001213641 | 0.112088394 |
| PWY.622..starch.biosynthesis | covid_severity | Severe | -1.90 | 0.57 | 241 | 73 | 0.001242247 | 0.113114497 |
| CENTFERM.PWY..pyruvate.fermentation.to.butanoate | covid_severity | Severe | -1.73 | 0.53 | 241 | 162 | 0.001329359 | 0.11773026 |
| PWY.6590..superpathway.of.Clostridium.acetobutylicum.acidogenic.fermentation | covid_severity | Severe | -1.72 | 0.52 | 241 | 162 | 0.001321942 | 0.11773026 |
| ARG.POLYAMINE.SYN..superpathway.of.arginine.and.polyamine.biosynthesis | covid_severity | Severe | -2.12 | 0.65 | 241 | 182 | 0.001388061 | 0.121267785 |
| P441.PWY..superpathway.of.N.acetylneuraminate.degradation | covid_severity | Severe | -1.34 | 0.41 | 241 | 208 | 0.001461193 | 0.124297514 |
| PWY.5505..L.glutamate.and.L.glutamine.biosynthesis | time_from_admit | time_from_admit | -0.05 | 0.02 | 241 | 177 | 0.00144588 | 0.124297514 |
| PWY.7211..superpathway.of.pyrimidine.deoxyribonucleotides.de.novo.biosynthesis | race | Black | -1.33 | 0.41 | 241 | 228 | 0.001486413 | 0.124555595 |
| PWY.7400..L.arginine.biosynthesis.IV..archaebacteria. | covid_severity | Severe | -1.35 | 0.42 | 241 | 233 | 0.001502759 | 0.124555595 |
| ARGSYN.PWY..L.arginine.biosynthesis.I..via.L.ornithine. | covid_severity | Severe | -1.19 | 0.37 | 241 | 236 | 0.001747435 | 0.136110415 |
| GLCMANNANAUT.PWY..superpathway.of.N.acetylglucosamine..N.acetylmannosamine.and.N.acetylneuraminate.degradation | covid_severity | Severe | -1.22 | 0.38 | 241 | 228 | 0.001729056 | 0.136110415 |
| POLYAMSYN.PWY..superpathway.of.polyamine.biosynthesis.I | covid_severity | Severe | -2.11 | 0.66 | 241 | 182 | 0.001743481 | 0.136110415 |
| PWY.5136..fatty.acid..beta..oxidation.II..peroxisome. | charlson | charlson | 0.81 | 0.25 | 241 | 209 | 0.001737465 | 0.136110415 |
| PWY.7392..taxadiene.biosynthesis..engineered. | read_depth | read_depth | 0.83 | 0.26 | 241 | 98 | 0.001699588 | 0.136110415 |
| X7ALPHADEHYDROX.PWY..cholate.degradation..bacteria..anaerobic. | read_depth | read_depth | 0.64 | 0.20 | 241 | 69 | 0.001908179 | 0.137609774 |
| GLUDEG.I.PWY..GABA.shunt | covid_severity | Severe | -1.89 | 0.60 | 241 | 190 | 0.001868302 | 0.137609774 |
| NONMEVIPP.PWY..methylerythritol.phosphate.pathway.I | time_from_admit | time_from_admit | -0.06 | 0.02 | 241 | 102 | 0.001915862 | 0.137609774 |
| PROTOCATECHUATE.ORTHO.CLEAVAGE.PWY..protocatechuate.degradation.II..ortho.cleavage.pathway. | time_from_admit | time_from_admit | 0.04 | 0.01 | 241 | 58 | 0.00189055 | 0.137609774 |
| PWY.5677..succinate.fermentation.to.butanoate | read_depth | read_depth | 0.59 | 0.19 | 241 | 127 | 0.001899118 | 0.137609774 |
| PWY.6531..mannitol.cycle | read_depth | read_depth | 0.71 | 0.23 | 241 | 217 | 0.001936967 | 0.137609774 |
| PWY.7209..superpathway.of.pyrimidine.ribonucleosides.degradation | on_abx | TRUE | -1.59 | 0.50 | 241 | 90 | 0.0019051 | 0.137609774 |
| SULFATE.CYS.PWY..superpathway.of.sulfate.assimilation.and.cysteine.biosynthesis | covid_severity | Severe | -1.91 | 0.61 | 241 | 187 | 0.001925013 | 0.137609774 |
| FUCCAT.PWY..fucose.degradation | covid_severity | Severe | -1.84 | 0.59 | 241 | 209 | 0.002102985 | 0.144636168 |
| PWY.5345..superpathway.of.L.methionine.biosynthesis..by.sulfhydrylation. | covid_severity | Severe | -1.78 | 0.57 | 241 | 186 | 0.002095594 | 0.144636168 |
| PWY66.388..fatty.acid..alpha..oxidation.III | age | age | 0.64 | 0.20 | 241 | 43 | 0.002088625 | 0.144636168 |
| PWY.4722..creatinine.degradation.II | read_depth | read_depth | 0.40 | 0.13 | 241 | 81 | 0.002132703 | 0.14513604 |
| NONMEVIPP.PWY..methylerythritol.phosphate.pathway.I | read_depth | read_depth | 0.87 | 0.28 | 241 | 102 | 0.002165594 | 0.145336444 |
| PWY.6270..isoprene.biosynthesis.I | time_from_admit | time_from_admit | -0.06 | 0.02 | 241 | 102 | 0.00220402 | 0.145336444 |
| PWY.6396..superpathway.of.2.3.butanediol.biosynthesis | male | yes | -0.89 | 0.28 | 241 | 53 | 0.00222557 | 0.145336444 |
| PWY66.367..ketogenesis | read_depth | read_depth | 0.53 | 0.17 | 241 | 113 | 0.002186746 | 0.145336444 |
| HEXITOLDEGSUPER.PWY..superpathway.of.hexitol.degradation..bacteria. | covid_severity | Severe | -0.98 | 0.32 | 241 | 229 | 0.002314241 | 0.149615652 |
| FAO.PWY..fatty.acid..beta..oxidation.I | charlson | charlson | 0.79 | 0.25 | 241 | 208 | 0.002569115 | 0.164448766 |
| PWY.6318..L.phenylalanine.degradation.IV..mammalian..via.side.chain. | race | Other | 2.91 | 0.94 | 241 | 173 | 0.002700174 | 0.169481775 |
| PWY.7013..L.1.2.propanediol.degradation | covid_severity | Severe | -1.92 | 0.63 | 241 | 195 | 0.002698989 | 0.169481775 |
| PWY.7332..superpathway.of.UDP.N.acetylglucosamine.derived.O.antigen.building.blocks.biosynthesis | tx_remdesivir | 1 | 1.97 | 0.65 | 241 | 160 | 0.00276303 | 0.171759485 |
| POLYAMINSYN3.PWY..superpathway.of.polyamine.biosynthesis.II | race | Black | 2.06 | 0.67 | 241 | 88 | 0.002926354 | 0.176811953 |
| PWY.5121..superpathway.of.geranylgeranyl.diphosphate.biosynthesis.II..via.MEP. | read_depth | read_depth | 0.77 | 0.25 | 241 | 101 | 0.002904144 | 0.176811953 |
| PWY.7245..superpathway.NAD.NADP...NADH.NADPH.interconversion..yeast. | time_from_admit | time_from_admit | 0.03 | 0.01 | 241 | 25 | 0.002889644 | 0.176811953 |
| PWY.6270..isoprene.biosynthesis.I | read_depth | read_depth | 0.87 | 0.29 | 241 | 102 | 0.003005882 | 0.179935425 |
| PWY.5100..pyruvate.fermentation.to.acetate.and.lactate.II | covid_severity | Severe | -0.99 | 0.33 | 241 | 238 | 0.003160813 | 0.187302168 |
| PWY.7279..aerobic.respiration.II..cytochrome.c...yeast. | charlson | charlson | 1.04 | 0.34 | 241 | 116 | 0.003186889 | 0.187302168 |
| COBALSYN.PWY..adenosylcobalamin.salvage.from.cobinamide.I | covid_severity | Severe | -1.64 | 0.55 | 241 | 216 | 0.003246255 | 0.189072436 |
| TCA..TCA.cycle.I..prokaryotic. | covid_severity | Severe | -1.68 | 0.56 | 241 | 214 | 0.003334304 | 0.192466744 |
| CITRULBIO.PWY..L.citrulline.biosynthesis | tx_remdesivir | 1 | 1.28 | 0.43 | 241 | 233 | 0.003528887 | 0.196795855 |
| PWY.5121..superpathway.of.geranylgeranyl.diphosphate.biosynthesis.II..via.MEP. | time_from_admit | time_from_admit | -0.05 | 0.02 | 241 | 101 | 0.003531063 | 0.196795855 |
| PWY.6182..superpathway.of.salicylate.degradation | time_from_admit | time_from_admit | 0.03 | 0.01 | 241 | 44 | 0.003465901 | 0.196795855 |
| PWY.7560..methylerythritol.phosphate.pathway.II | read_depth | read_depth | 0.73 | 0.25 | 241 | 102 | 0.003509764 | 0.196795855 |
| POLYAMINSYN3.PWY..superpathway.of.polyamine.biosynthesis.II | covid_severity | Severe | -1.61 | 0.54 | 241 | 88 | 0.003577643 | 0.197632206 |
| PWY.6263..superpathway.of.menaquinol.8.biosynthesis.II | tx_remdesivir | 1 | 2.07 | 0.70 | 241 | 140 | 0.003607208 | 0.197632206 |
| CODH.PWY..reductive.acetyl.coenzyme.A.pathway | time_from_admit | time_from_admit | 0.04 | 0.01 | 241 | 90 | 0.003671844 | 0.199482959 |
| P124.PWY..Bifidobacterium.shunt | covid_severity | Severe | -1.89 | 0.64 | 241 | 132 | 0.003752651 | 0.202174061 |
| PWY.7198..pyrimidine.deoxyribonucleotides.de.novo.biosynthesis.IV | read_depth | read_depth | 0.41 | 0.14 | 241 | 235 | 0.003815018 | 0.203835477 |
| PWY.7315..dTDP.N.acetylthomosamine.biosynthesis | covid_severity | Severe | -1.96 | 0.67 | 241 | 181 | 0.003933379 | 0.208436823 |
| GLUTORN.PWY..L.ornithine.biosynthesis | covid_severity | Severe | -1.20 | 0.41 | 241 | 236 | 0.004082988 | 0.21117213 |
| PWY.5028..L.histidine.degradation.II | on_abx | TRUE | 1.36 | 0.47 | 241 | 66 | 0.004068912 | 0.21117213 |
| PWY.7200..superpathway.of.pyrimidine.deoxyribonucleoside.salvage | read_depth | read_depth | -0.44 | 0.15 | 241 | 39 | 0.004070673 | 0.21117213 |
| PWY.6507..4.deoxy.L.threo.hex.4.enopyranuronate.degradation | time_from_admit | time_from_admit | 0.03 | 0.01 | 241 | 226 | 0.004337952 | 0.222578261 |
| PWY.3781..aerobic.respiration.I..cytochrome.c. | charlson | charlson | 1.08 | 0.37 | 241 | 170 | 0.004528117 | 0.227297606 |
| PWY.7392..taxadiene.biosynthesis..engineered. | time_from_admit | time_from_admit | -0.05 | 0.02 | 241 | 98 | 0.004535405 | 0.227297606 |
| SO4ASSIM.PWY..sulfate.reduction.I..assimilatory. | covid_severity | Severe | -1.82 | 0.63 | 241 | 190 | 0.004504012 | 0.227297606 |
| HEXITOLDEGSUPER.PWY..superpathway.of.hexitol.degradation..bacteria. | time_from_admit | time_from_admit | 0.02 | 0.01 | 241 | 229 | 0.004606808 | 0.229100095 |
| PROPFERM.PWY..L.alanine.fermentation.to.propanoate.and.acetate | read_depth | read_depth | 0.22 | 0.08 | 241 | 42 | 0.004864033 | 0.229532634 |
| PRPP.PWY..superpathway.of.histidine..purine..and.pyrimidine.biosynthesis | covid_severity | Severe | -1.05 | 0.37 | 241 | 214 | 0.004772472 | 0.229532634 |
| PWY.5088..L.glutamate.degradation.VIII..to.propanoate. | covid_severity | Severe | -0.99 | 0.35 | 241 | 28 | 0.00483793 | 0.229532634 |
| PWY.5417..catechol.degradation.III..ortho.cleavage.pathway. | time_from_admit | time_from_admit | 0.03 | 0.01 | 241 | 49 | 0.004823022 | 0.229532634 |
| PWY.5431..aromatic.compounds.degradation.via..beta..ketoadipate | time_from_admit | time_from_admit | 0.03 | 0.01 | 241 | 49 | 0.004823022 | 0.229532634 |
| PWY.7268..NAD.NADP.NADH.NADPH.cytosolic.interconversion..yeast. | time_from_admit | time_from_admit | 0.03 | 0.01 | 241 | 42 | 0.004689052 | 0.229532634 |
| PWY3O.19..ubiquinol.6.biosynthesis.from.4.hydroxybenzoate..eukaryotic. | covid_severity | Severe | 1.10 | 0.39 | 241 | 38 | 0.004744333 | 0.229532634 |
| PWY.7210..pyrimidine.deoxyribonucleotides.biosynthesis.from.CTP | charlson | charlson | 0.88 | 0.30 | 241 | 191 | 0.004936598 | 0.231268864 |
| GALACTUROCAT.PWY..D.galacturonate.degradation.I | read_depth | read_depth | 0.47 | 0.17 | 241 | 228 | 0.005019797 | 0.231807034 |
| P125.PWY..superpathway.of..R.R..butanediol.biosynthesis | ethnicity | Hispanic | -1.90 | 0.66 | 241 | 156 | 0.00501123 | 0.231807034 |
| PWY.7456..mannan.degradation | tx_remdesivir | 1 | 1.82 | 0.64 | 241 | 201 | 0.005071322 | 0.232525496 |
| CITRULBIO.PWY..L.citrulline.biosynthesis | ethnicity | Hispanic | -1.21 | 0.42 | 241 | 233 | 0.005141712 | 0.2336575 |
| PWY.6318..L.phenylalanine.degradation.IV..mammalian..via.side.chain. | ethnicity | Hispanic | -1.95 | 0.68 | 241 | 173 | 0.005168294 | 0.2336575 |
| GLUCONEO.PWY..gluconeogenesis.I | covid_severity | Severe | -0.97 | 0.34 | 241 | 233 | 0.005210725 | 0.23393983 |
| PWY.7456..mannan.degradation | time_from_admit | time_from_admit | -0.04 | 0.01 | 241 | 201 | 0.005265382 | 0.234763429 |
| PWY.7013..L.1.2.propanediol.degradation | read_depth | read_depth | 0.72 | 0.26 | 241 | 195 | 0.005433019 | 0.240578527 |
| PWY.6606..guanosine.nucleotides.degradation.II | covid_severity | Severe | -1.22 | 0.43 | 241 | 229 | 0.005472113 | 0.240661306 |
| PWY.6163..chorismate.biosynthesis.from.3.dehydroquinate | time_from_admit | time_from_admit | -0.01 | 0.00 | 241 | 241 | 0.005570249 | 0.243322017 |
| DTDPRHAMSYN.PWY..dTDP.L.rhamnose.biosynthesis.I | charlson | charlson | -0.22 | 0.08 | 241 | 240 | 0.005753383 | 0.247970817 |
| PWY.5005..biotin.biosynthesis.II | read_depth | read_depth | 0.67 | 0.24 | 241 | 153 | 0.005743685 | 0.247970817 |
| Results with FDR-corrected p-value <0.25 shown. | | | | | | | | |

**Table S4. Sensitivity analyses for differentially abundant species**

| **feature** | **coef** | **qval** | **coef.30days** | **qval.30days** | **coef.noabx** | **qval.noabx** |
| --- | --- | --- | --- | --- | --- | --- |
| *Eubacterium_eligens* | -5.5832084 | 5.15E-07 | -4.709453875 | 0.000219948 | -4.10508691 | 0.001685878 |
| *Eubacterium_hallii* | -5.0163338 | 5.15E-07 | -4.207716881 | 0.000173656 | -5.29525079 | 0.000950389 |
| *Roseburia_hominis* | -5.8666776 | 6.20E-07 | -5.986112293 | 0.000173656 | -7.46775885 | 2.04E-05 |
| *Anaerostipes_hadrus* | -5.1071009 | 1.20E-06 | -4.829775781 | 0.000173656 | -5.93631609 | 1.12E-05 |
| *Fusicatenibacter_saccharivorans* | -5.6631916 | 1.51E-06 | -5.032718696 | 0.000353203 | -5.06910737 | 0.000273871 |
| *Adlercreutzia_equolifaciens* | -5.0369745 | 1.86E-06 | -5.011172763 | 0.000173656 | -5.52795456 | 0.00150899 |
| *Actinomyces_odontolyticus* | -2.4393832 | 1.86E-06 | -2.193669126 | 0.000858301 | -2.07438282 | 0.020284387 |
| *Agathobaculum_butyriciproducens* | -3.4863789 | 1.86E-06 | -3.150186076 | 0.000767196 | -5.02187341 | 3.37E-05 |
| *Asaccharobacter_celatus* | -5.4246427 | 1.93E-06 | -5.130862112 | 0.000173656 | -4.32835485 | 0.002053847 |
| *Clostridium_leptum* | -3.8858413 | 7.33E-06 | -3.487031074 | 0.000389232 | -3.5668137 | 0.002195914 |
| *Roseburia_intestinalis* | -4.0308729 | 1.36E-05 | -3.564274001 | 0.003525944 | -4.03491937 | 0.012155573 |
| *Blautia_wexlerae* | -4.9995534 | 1.65E-05 | -4.110112837 | 0.007940355 | -5.31690364 | 0.00218463 |
| *Streptococcus_parasanguinis* | -4.1748186 | 1.73E-05 | -3.559321151 | 0.003751271 | -4.13230696 | 0.002325936 |
| *Dorea_formicigenerans* | -4.3654805 | 4.65E-05 | -3.543798641 | 0.010522257 | -3.73927967 | 0.00036882 |
| *Eubacterium_siraeum* | -4.4143572 | 4.91E-05 | -4.152861829 | 0.003574154 | -5.36330079 | 0.00218463 |
| *Gemmiger_formicilis* | -3.9314529 | 5.00E-05 | -3.076516703 | 0.016603643 | -5.57903098 | 0.000128268 |
| *Enterorhabdus_caecimuris* | -2.546151 | 7.87E-05 | -2.363088677 | 0.006279308 | -2.79860848 | 0.024840283 |
| *Eubacterium_rectale* | -4.3717115 | 0.0001214 | -4.183504605 | 0.004793083 | -4.57357912 | 0.007919486 |
| *Actinomyces_sp_HMSC035G02* | -1.9563721 | 0.00012776 | -1.720751908 | 0.007630607 | -2.44461722 | 0.013992945 |
| *Oscillibacter_sp_57_20* | -2.6840001 | 0.00017097 | -2.358833338 | 0.010522257 | -3.42602186 | 0.013992945 |
| *Ruminococcus_bicirculans* | -3.5330127 | 0.00024464 | -3.032096893 | 0.016603643 | -4.21034552 | 0.012155573 |
| *Ruminococcus_torques* | -3.800818 | 0.00028714 | -3.17054285 | 0.024297086 | -5.22351322 | 0.002325936 |
| *Streptococcus_mitis* | -2.4106662 | 0.00035473 | -2.190097099 | 0.011273922 | -3.2328794 | 0.004980108 |
| *Blautia_sp_CAG_257* | -3.2898274 | 0.00040078 | -3.187072874 | 0.010656317 | -3.59792347 | 0.027859811 |
| *Streptococcus_salivarius* | -3.1764579 | 0.00047703 | -2.484767187 | 0.04410097 | -4.53658893 | 0.000373589 |
| *Collinsella_stercoris* | -2.8489212 | 0.00049699 | -2.582680528 | 0.024059204 | -2.81844006 | 0.013992945 |
| *Rothia_mucilaginosa* | -2.5275676 | 0.00049699 | -2.408516156 | 0.009064483 | -2.56265832 | 0.033000275 |
| *Roseburia_inulinivorans* | -3.256794 | 0.00050344 | -2.328033351 | 0.080385173 | -4.57182392 | 0.00896313 |
| *Actinomyces_sp_HPA0247* | -1.8044026 | 0.00143674 | -1.9411488 | 0.007630607 | -1.66977378 | 0.140261906 |
| *Streptococcus_gordonii* | -1.7396782 | 0.00222911 | -1.415013998 | 0.07572447 | -1.22482521 | 0.323008026 |
| *Streptococcus_oralis* | -2.055192 | 0.00265595 | -1.677750198 | 0.081898857 | -2.08269493 | 0.180367695 |
| *Monoglobus_pectinilyticus* | -2.7846876 | 0.00332723 | -2.768115813 | 0.019941865 | -2.44236752 | 0.025832966 |
| *Streptococcus_thermophilus* | -2.9386325 | 0.00350101 | -3.188965292 | 0.006683044 | -3.63901104 | 0.014099678 |
| *Actinomyces_oris* | -1.8466171 | 0.00494199 | -1.36271487 | 0.143984074 | -3.08580843 | 0.00218463 |
| *Clostridium_sp_CAG_58* | -2.666553 | 0.00623796 | -2.334619578 | 0.065475045 | -4.34964937 | 0.014099678 |
| *Dorea_longicatena* | -3.5092287 | 0.00628752 | -2.770933101 | 0.144596262 | -3.09293676 | 0.070275367 |
| *Collinsella_aerofaciens* | -4.7146677 | 0.01002521 | -3.581874748 | 0.100906426 | -6.39980761 | 0.009841753 |
| *Coprococcus_catus* | -2.7206636 | 0.01022847 | -1.966648181 | 0.211809025 | -3.56191912 | 0.077977987 |
| *Blautia_obeum* | -3.2877517 | 0.01334091 | -3.164097121 | 0.065874308 | -2.81525322 | 0.093983617 |
| *Roseburia_faecis* | -2.5798958 | 0.01807029 | -2.194280012 | 0.143984074 | -3.38698873 | 0.150693353 |
| *Bifidobacterium_adolescentis* | -3.2683674 | 0.01928692 | -1.9213695 | 0.215543859 | -4.89639363 | 0.075946398 |
| *Eubacterium_ramulus* | -2.0819377 | 0.02351652 | -1.964193997 | 0.108101739 | -1.61503252 | 0.436327891 |
| *Candida_albicans* | 2.8506306 | 0.02456256 | 3.026150378 | 0.039155999 | 2.022195096 | 0.274920856 |
| *Ruminococcus_gnavus* | -4.3489008 | 0.02600178 | -3.240639184 | 0.088490298 | -3.74304546 | 0.372427646 |
| *Ruminococcus_bromii* | -3.7464714 | 0.03436974 | -1.765881687 | 0.271008798 | -5.78232498 | 0.006144659 |
| *Coprococcus_comes* | -2.8811076 | 0.04108115 | -2.251647477 | 0.265172592 | -3.22164639 | 0.076938624 |
| *Blautia_hydrogenotrophica* | -2.9820506 | 0.04964429 | -2.798760243 | 0.144596262 | -3.44615281 | 0.04613349 |
| *Lactococcus_lactis* | -1.1835325 | 0.04964429 | -1.280625036 | 0.010522257 | -0.71967682 | 0.547699527 |

**Table S5. Node and network-specific information**

| **Moderate Network** | | | | | | | | | |
| --- | --- | --- | --- | --- | --- | --- | --- | --- | --- |
| **Cluster Color** | **Species** | **Edge Count** | **Degree** | **Betweeness** | **Closeness** | **Eigenvector Centrality** | **Hub Taxon (> 0.8)** | **Hub in Both Networks?** | **Singleton in Both Networks?** |
| Sky blue | *Gordonibacter pamelaeae* | 23 | 0.17 | 0.11 | 1.00 | 0.14 | Yes | Yes |  |
| Sky blue | *Alistipes putredinis* | 22 | 0.16 | 0.08 | 1.00 | 0.15 | Yes |  |  |
| Sky blue | *Odoribacter splanchnicus* | 18 | 0.13 | 0.07 | 0.98 | 0.26 | Yes | Yes |  |
| Sky blue | *Eisenbergiella tayi* | 17 | 0.13 | 0.02 | 0.93 | 0.09 |  |  |  |
| Sky blue | *Bacteroides ovatus* | 14 | 0.10 | 0.01 | 0.94 | 0.27 |  |  |  |
| Sky blue | *Alistipes shahii* | 14 | 0.10 | 0.04 | 0.91 | 0.24 | Yes | Yes |  |
| Sky blue | *Methanobrevibacter smithii* | 13 | 0.10 | 0.06 | 0.84 | 0.09 |  |  |  |
| Sky blue | *Blautia obeum* | 13 | 0.10 | 0.02 | 0.88 | 0.36 |  |  |  |
| Sky blue | *Alistipes finegoldii* | 12 | 0.09 | 0.02 | 0.85 | 0.06 |  |  |  |
| Sky blue | *Bacteroides salyersiae* | 11 | 0.08 | 0.03 | 0.82 | 0.06 |  |  |  |
| Sky blue | *Alistipes indistinctus* | 11 | 0.08 | 0.03 | 0.87 | 0.11 |  |  |  |
| Sky blue | *Butyricimonas synergistica* | 11 | 0.08 | 0.03 | 0.83 | 0.08 |  |  |  |
| Sky blue | *Butyricimonas virosa* | 10 | 0.07 | 0.02 | 0.82 | 0.06 |  |  |  |
| Sky blue | *Bacteroides massiliensis* | 10 | 0.07 | 0.01 | 0.79 | 0.05 | Yes |  |  |
| Sky blue | *Dorea sp CAG 317* | 9 | 0.07 | 0.03 | 0.77 | 0.03 |  |  |  |
| Sky blue | *Monoglobus pectinilyticus* | 9 | 0.07 | 0.01 | 0.76 | 0.04 |  |  |  |
| Sky blue | *Adlercreutzia equolifaciens* | 9 | 0.07 | 0.01 | 0.89 | 0.06 |  |  |  |
| Sky blue | *Eggerthella lenta* | 8 | 0.06 | 0.00 | 0.84 | 0.05 |  |  |  |
| Sky blue | *Asaccharobacter celatus* | 8 | 0.06 | 0.01 | 0.88 | 0.06 |  |  |  |
| Sky blue | *Firmicutes bacterium CAG 145* | 8 | 0.06 | 0.02 | 0.79 | 0.03 |  |  |  |
| Sky blue | *Harryflintia acetispora* | 7 | 0.05 | 0.00 | 0.75 | 0.03 |  |  |  |
| Sky blue | *Clostridium methylpentosum* | 6 | 0.04 | 0.01 | 0.69 | 0.01 |  |  |  |
| Sky blue | *Bacteroides cellulosilyticus* | 6 | 0.04 | 0.00 | 0.73 | 0.02 |  |  |  |
| Sky blue | *Holdemania filiformis* | 5 | 0.04 | 0.00 | 0.74 | 0.04 |  |  |  |
| Sky blue | *Clostridium bolteae* | 5 | 0.04 | 0.02 | 0.72 | 0.05 |  |  |  |
| Sky blue | *Clostridium hylemonae* | 5 | 0.04 | 0.01 | 0.68 | 0.01 |  |  |  |
| Sky blue | *Clostridium lavalense* | 4 | 0.03 | 0.00 | 0.71 | 0.04 |  |  |  |
| Sky blue | *Bacteroides finegoldii* | 4 | 0.03 | 0.00 | 0.67 | 0.01 |  |  |  |
| Sky blue | *Catabacter hongkongensis* | 4 | 0.03 | 0.01 | 0.70 | 0.02 |  |  |  |
| Sky blue | *Anaerofustis stercorihominis* | 3 | 0.02 | 0.00 | 0.66 | 0.01 |  |  |  |
| Sky blue | *Eubacterium siraeum* | 3 | 0.02 | 0.00 | 0.68 | 0.03 |  |  |  |
| Sky blue | *Intestinimonas butyriciproducens* | 2 | 0.01 | 0.00 | 0.61 | 0.01 |  |  |  |
| Sky blue | *Bacteroides dorei* | 2 | 0.01 | 0.00 | 0.67 | 0.03 |  |  |  |
| Sky blue | *Bacteroides fragilis* | 2 | 0.01 | 0.00 | 0.69 | 0.05 |  |  |  |
| Sky blue | *Bacteroides faecis* | 2 | 0.01 | 0.02 | 0.54 | 0.00 |  |  |  |
| Sky blue | *Bacteroides nordii* | 2 | 0.01 | 0.00 | 0.65 | 0.01 |  |  |  |
| Sky blue | *Eubacterium limosum* | 2 | 0.01 | 0.02 | 0.57 | 0.00 |  |  |  |
| Sky blue | *Streptococcus thermophilus* | 1 | 0.01 | 0.00 | 0.54 | 0.00 |  |  |  |
| Sky blue | *Dielma fastidiosa* | 1 | 0.01 | 0.00 | 0.56 | 0.00 |  |  |  |
| Sky blue | *Eubacterium callanderi* | 1 | 0.01 | 0.00 | 0.43 | 0.00 |  |  |  |
| Sky blue | *Bacteroides faecis CAG 32* | 1 | 0.01 | 0.00 | 0.43 | 0.00 |  |  |  |
| Sky blue | *Bacteroides caccae* | 1 | 0.01 | 0.00 | 0.56 | 0.00 |  |  |  |
| Sky blue | *Clostridium bolteae CAG 59* | 1 | 0.01 | 0.00 | 0.53 | 0.00 |  |  |  |
| Purple | *Bacteroides uniformis* | 25 | 0.18 | 0.15 | 1.07 | 0.32 | Yes |  |  |
| Purple | *Eisenbergiella massiliensis* | 20 | 0.15 | 0.06 | 0.94 | 0.09 |  |  |  |
| Purple | *Blautia sp CAG 257* | 17 | 0.13 | 0.01 | 0.83 | 0.14 |  |  |  |
| Purple | *Sellimonas intestinalis* | 16 | 0.12 | 0.02 | 0.84 | 0.07 |  |  |  |
| Purple | *Blautia producta* | 15 | 0.11 | 0.03 | 0.82 | 0.11 |  |  |  |
| Purple | *Streptococcus salivarius* | 12 | 0.09 | 0.06 | 0.89 | 0.19 |  |  |  |
| Purple | *Anaerotruncus colihominis* | 11 | 0.08 | 0.05 | 0.83 | 0.04 |  |  |  |
| Purple | *Streptococcus gordonii* | 11 | 0.08 | 0.02 | 0.82 | 0.06 |  |  |  |
| Purple | *Parabacteroides distasonis* | 11 | 0.08 | 0.04 | 0.87 | 0.07 |  |  |  |
| Purple | *Bacteroides thetaiotaomicron* | 11 | 0.08 | 0.01 | 0.89 | 0.24 |  |  |  |
| Purple | *Clostridium scindens* | 11 | 0.08 | 0.04 | 0.78 | 0.07 |  |  |  |
| Purple | *Blautia hydrogenotrophica* | 10 | 0.07 | 0.00 | 0.78 | 0.04 |  |  |  |
| Purple | *Bacteroides stercoris* | 10 | 0.07 | 0.02 | 0.83 | 0.05 |  |  |  |
| Purple | *Lawsonibacter asaccharolyticus* | 9 | 0.07 | 0.00 | 0.84 | 0.07 |  |  |  |
| Purple | *Clostridium symbiosum* | 9 | 0.07 | 0.00 | 0.81 | 0.05 |  |  |  |
| Purple | *Anaerostipes caccae* | 9 | 0.07 | 0.00 | 0.77 | 0.06 |  |  |  |
| Purple | *Clostridium innocuum* | 9 | 0.07 | 0.01 | 0.74 | 0.05 |  |  |  |
| Purple | *Parabacteroides merdae* | 8 | 0.06 | 0.01 | 0.84 | 0.10 |  |  |  |
| Purple | *Ruminococcus gnavus* | 8 | 0.06 | 0.01 | 0.72 | 0.03 |  |  |  |
| Purple | *Streptococcus parasanguinis* | 8 | 0.06 | 0.01 | 0.72 | 0.03 |  |  |  |
| Purple | *Flavonifractor plautii* | 7 | 0.05 | 0.00 | 0.81 | 0.08 |  |  |  |
| Purple | *Ruthenibacterium lactatiformans* | 7 | 0.05 | 0.02 | 0.78 | 0.04 |  |  |  |
| Purple | *Clostridium spiroforme* | 6 | 0.04 | 0.00 | 0.73 | 0.02 |  |  |  |
| Purple | *Streptococcus vestibularis* | 6 | 0.04 | 0.01 | 0.71 | 0.02 |  |  |  |
| Purple | *Actinomyces odontolyticus* | 6 | 0.04 | 0.00 | 0.72 | 0.06 |  |  |  |
| Purple | *Bacteroides vulgatus* | 6 | 0.04 | 0.01 | 0.78 | 0.06 |  |  |  |
| Purple | *Actinomyces oris* | 5 | 0.04 | 0.00 | 0.66 | 0.02 |  |  |  |
| Purple | *Rothia mucilaginosa* | 5 | 0.04 | 0.00 | 0.67 | 0.02 |  |  |  |
| Purple | *Bacteroides xylanisolvens* | 4 | 0.03 | 0.01 | 0.69 | 0.03 |  |  |  |
| Purple | *Clostridium leptum* | 3 | 0.02 | 0.02 | 0.67 | 0.02 |  |  |  |
| Purple | *Clostridium clostridioforme* | 3 | 0.02 | 0.00 | 0.61 | 0.00 |  |  |  |
| Purple | *Erysipelatoclostridium ramosum* | 3 | 0.02 | 0.00 | 0.68 | 0.01 |  |  |  |
| Purple | *Anaerotignum lactatifermentans* | 3 | 0.02 | 0.00 | 0.70 | 0.02 |  |  |  |
| Purple | *Bilophila wadsworthia* | 3 | 0.02 | 0.00 | 0.70 | 0.02 |  |  |  |
| Purple | *Clostridium citroniae* | 2 | 0.01 | 0.00 | 0.66 | 0.06 |  |  |  |
| Purple | *Ruminococcaceae bacterium D16* | 2 | 0.01 | 0.02 | 0.57 | 0.00 |  |  |  |
| Purple | *Clostridium asparagiforme* | 1 | 0.01 | 0.00 | 0.43 | 0.00 |  |  |  |
| Purple | *Klebsiella pneumoniae* | 1 | 0.01 | 0.00 | 0.42 | 0.00 |  |  |  |
| Gold | *Blautia coccoides* | 5 | 0.04 | 0.03 | 0.60 | 0.01 |  |  |  |
| Gold | *Enterococcus faecium* | 4 | 0.03 | 0.01 | 0.61 | 0.03 |  |  |  |
| Gold | *Lactobacillus rhamnosus* | 4 | 0.03 | 0.01 | 0.54 | 0.00 |  |  |  |
| Gold | *Enterococcus faecalis* | 2 | 0.01 | 0.00 | 0.47 | 0.00 |  |  |  |
| Gold | *Barnesiella intestinihominis* | 1 | 0.01 | 0.00 | 0.39 | 0.00 |  |  |  |
| Pink | *Lactobacillus fermentum* | 9 | 0.07 | 0.03 | 0.63 | 0.06 |  |  |  |
| Pink | *Veillonella parvula* | 7 | 0.05 | 0.02 | 0.62 | 0.00 |  |  |  |
| Pink | *Streptococcus anginosus group* | 6 | 0.04 | 0.02 | 0.70 | 0.01 |  |  |  |
| Pink | *Parabacteroides goldsteinii* | 5 | 0.04 | 0.01 | 0.68 | 0.01 |  |  |  |
| Pink | *Lactobacillus paragasseri* | 5 | 0.04 | 0.00 | 0.56 | 0.01 |  |  |  |
| Pink | *Candida albicans* | 5 | 0.04 | 0.01 | 0.63 | 0.03 |  |  |  |
| Pink | *Bifidobacterium longum* | 4 | 0.03 | 0.01 | 0.68 | 0.05 |  |  |  |
| Pink | *Lactobacillus gasseri* | 4 | 0.03 | 0.00 | 0.53 | 0.00 |  |  |  |
| Pink | *Bifidobacterium breve* | 4 | 0.03 | 0.00 | 0.53 | 0.00 |  |  |  |
| Pink | *Phascolarctobacterium faecium* | 3 | 0.02 | 0.00 | 0.66 | 0.01 |  |  |  |
| Green | *Faecalibacterium prausnitzii* | 28 | 0.21 | 0.05 | 1.04 | 0.96 | Yes |  |  |
| Green | *Dorea formicigenerans* | 27 | 0.20 | 0.03 | 1.04 | 1.00 | Yes |  |  |
| Green | *Fusicatenibacter saccharivorans* | 25 | 0.18 | 0.02 | 0.97 | 0.91 |  |  |  |
| Green | *Anaerostipes hadrus* | 23 | 0.17 | 0.03 | 1.00 | 0.85 | Yes |  |  |
| Green | *Gemmiger formicilis* | 22 | 0.16 | 0.00 | 0.94 | 0.89 |  |  |  |
| Green | *Ruminococcus torques* | 21 | 0.15 | 0.02 | 0.91 | 0.74 |  |  |  |
| Green | *Eubacterium hallii* | 21 | 0.15 | 0.05 | 1.00 | 0.78 | Yes | Yes |  |
| Green | *Coprococcus catus* | 21 | 0.15 | 0.01 | 0.91 | 0.83 |  |  |  |
| Green | *Eubacterium rectale* | 21 | 0.15 | 0.04 | 0.97 | 0.76 | Yes |  |  |
| Green | *Blautia wexlerae* | 20 | 0.15 | 0.05 | 0.95 | 0.67 | Yes | Yes |  |
| Green | *Roseburia hominis* | 20 | 0.15 | 0.01 | 0.97 | 0.75 |  |  |  |
| Green | *Roseburia inulinivorans* | 20 | 0.15 | 0.01 | 0.92 | 0.81 |  |  |  |
| Green | *Coprococcus comes* | 20 | 0.15 | 0.01 | 0.89 | 0.80 |  |  |  |
| Green | *Oscillibacter sp 57 20* | 19 | 0.14 | 0.01 | 0.86 | 0.66 |  |  |  |
| Green | *Collinsella aerofaciens* | 17 | 0.13 | 0.02 | 0.91 | 0.62 |  |  |  |
| Green | *Oscillibacter sp CAG 241* | 16 | 0.12 | 0.01 | 0.84 | 0.47 |  |  |  |
| Green | *Dorea longicatena* | 16 | 0.12 | 0.00 | 0.89 | 0.68 |  |  |  |
| Green | *Agathobaculum butyriciproducens* | 15 | 0.11 | 0.00 | 0.84 | 0.52 |  |  |  |
| Green | *Eubacterium ramulus* | 15 | 0.11 | 0.00 | 0.85 | 0.67 |  |  |  |
| Green | *Eubacterium eligens* | 13 | 0.10 | 0.01 | 0.86 | 0.49 |  |  |  |
| Green | *Prevotella copri* | 13 | 0.10 | 0.00 | 0.77 | 0.39 |  |  |  |
| Green | *Clostridium sp CAG 58* | 12 | 0.09 | 0.00 | 0.86 | 0.37 |  |  |  |
| Green | *Roseburia intestinalis* | 10 | 0.07 | 0.00 | 0.78 | 0.41 |  |  |  |
| Green | *Collinsella stercoris* | 8 | 0.06 | 0.03 | 0.82 | 0.11 |  |  |  |
| Green | *Roseburia faecis* | 7 | 0.05 | 0.01 | 0.74 | 0.24 |  |  |  |
| Green | *Bifidobacterium adolescentis* | 6 | 0.04 | 0.00 | 0.74 | 0.14 |  |  |  |
| Green | *Holdemanella biformis* | 6 | 0.04 | 0.00 | 0.68 | 0.23 |  |  |  |
| Green | *Akkermansia muciniphila* | 3 | 0.02 | 0.00 | 0.71 | 0.08 |  |  |  |
| Green | *Ruminococcus bromii* | 3 | 0.02 | 0.00 | 0.68 | 0.12 |  |  |  |
| Green | *Firmicutes bacterium CAG 83* | 3 | 0.02 | 0.00 | 0.67 | 0.14 |  |  |  |
| Violet red | *Parasutterella excrementihominis* | 3 | 0.02 | 0.02 | 0.63 | 0.00 |  |  |  |
| Violet red | *Turicimonas muris* | 3 | 0.02 | 0.00 | 0.56 | 0.00 |  |  |  |
| Violet red | *Proteobacteria bacterium CAG 139* | 2 | 0.01 | 0.00 | 0.56 | 0.00 |  |  |  |
| Grey | *Pseudomonas aeruginosa group* | NA | 0.00 | 0.00 | 0.00 | 0.00 |  |  | Yes |
| Grey | *Hungatella hathewayi* | NA | 0.00 | 0.00 | 0.00 | 0.00 |  |  |  |
| Grey | *Escherichia coli* | NA | 0.00 | 0.00 | 0.00 | 0.00 |  |  |  |
| Grey | *Paraprevotella xylaniphila* | NA | 0.00 | 0.00 | 0.00 | 0.00 |  |  |  |
| Grey | *Coprobacillus cateniformis* | NA | 0.00 | 0.00 | 0.00 | 0.00 |  |  | Yes |
| Grey | *Faecalicatena orotica* | NA | 0.00 | 0.00 | 0.00 | 0.00 |  |  |  |
| Grey | *Acidaminococcus intestini* | NA | 0.00 | 0.00 | 0.00 | 0.00 |  |  | Yes |
| Grey | *Christensenella minuta* | NA | 0.00 | 0.00 | 0.00 | 0.00 |  |  |  |
| **Severe Network** | | | | | | | | | |
| **Cluster Color** | **Species** | **Edge Count** | **Degree** | **Betweeness** | **Closeness** | **Eigenvector Centrality** | **Hub Taxon (> 0.8)** | **Hub in Both Networks?** | **Singleton in Both Networks?** |
| Brick red | *Streptococcus salivarius* | 21 | 0.15 | 0.04 | 1.00 | 0.64 | Yes |  |  |
| Brick red | *Streptococcus parasanguinis* | 16 | 0.12 | 0.03 | 0.96 | 0.40 |  |  |  |
| Brick red | *Anaerotruncus colihominis* | 13 | 0.10 | 0.03 | 0.91 | 0.35 |  |  |  |
| Brick red | *Actinomyces oris* | 12 | 0.09 | 0.03 | 0.84 | 0.22 |  |  |  |
| Brick red | *Streptococcus vestibularis* | 11 | 0.08 | 0.00 | 0.86 | 0.32 |  |  |  |
| Brick red | *Eisenbergiella massiliensis* | 11 | 0.08 | 0.05 | 0.89 | 0.18 |  |  |  |
| Brick red | *Dorea sp CAG 317* | 9 | 0.07 | 0.01 | 0.83 | 0.21 |  |  |  |
| Brick red | *Actinomyces odontolyticus* | 9 | 0.07 | 0.00 | 0.83 | 0.25 |  |  |  |
| Brick red | *Streptococcus gordonii* | 7 | 0.05 | 0.00 | 0.78 | 0.19 |  |  |  |
| Brick red | *Anaerofustis stercorihominis* | 6 | 0.04 | 0.02 | 0.75 | 0.05 |  |  |  |
| Brick red | *Streptococcus anginosus group* | 5 | 0.04 | 0.00 | 0.72 | 0.06 |  |  |  |
| Brick red | *Oscillibacter sp 57 20* | 5 | 0.04 | 0.00 | 0.73 | 0.16 |  |  |  |
| Brick red | *Ruminococcaceae bacterium D16* | 5 | 0.04 | 0.00 | 0.72 | 0.11 |  |  |  |
| Brick red | *Monoglobus pectinilyticus* | 4 | 0.03 | 0.00 | 0.67 | 0.07 |  |  |  |
| Brick red | *Clostridium asparagiforme* | 3 | 0.02 | 0.04 | 0.64 | 0.01 |  |  |  |
| Brick red | *Blautia sp CAG 257* | 2 | 0.01 | 0.00 | 0.59 | 0.01 |  |  |  |
| Brick red | *Bacteroides faecis* | 2 | 0.01 | 0.02 | 0.49 | 0.00 |  |  |  |
| Brick red | *Rothia mucilaginosa* | 2 | 0.01 | 0.00 | 0.63 | 0.03 |  |  |  |
| Brick red | *Bacteroides faecis CAG 32* | 1 | 0.01 | 0.00 | 0.41 | 0.00 |  |  |  |
| Green | *Collinsella aerofaciens* | 29 | 0.21 | 0.07 | 1.10 | 0.95 | Yes |  |  |
| Green | *Roseburia inulinivorans* | 29 | 0.21 | 0.05 | 1.07 | 1.00 | Yes |  |  |
| Green | *Eubacterium hallii* | 26 | 0.19 | 0.10 | 1.08 | 0.83 | Yes | Yes |  |
| Green | *Blautia obeum* | 25 | 0.18 | 0.08 | 1.09 | 0.90 | Yes |  |  |
| Green | *Dorea longicatena* | 23 | 0.17 | 0.02 | 0.99 | 0.83 |  |  |  |
| Green | *Collinsella stercoris* | 22 | 0.16 | 0.03 | 1.01 | 0.78 |  |  |  |
| Green | *Ruminococcus torques* | 22 | 0.16 | 0.03 | 1.02 | 0.89 |  |  |  |
| Green | *Anaerostipes hadrus* | 22 | 0.16 | 0.01 | 1.00 | 0.85 |  |  |  |
| Green | *Coprococcus catus* | 22 | 0.16 | 0.01 | 0.98 | 0.85 |  |  |  |
| Green | *Blautia wexlerae* | 20 | 0.15 | 0.04 | 0.97 | 0.70 | Yes | Yes |  |
| Green | *Dorea formicigenerans* | 20 | 0.15 | 0.02 | 0.99 | 0.83 |  |  |  |
| Green | *Ruminococcus bromii* | 19 | 0.14 | 0.02 | 0.95 | 0.70 |  |  |  |
| Green | *Alistipes shahii* | 19 | 0.14 | 0.07 | 0.98 | 0.55 | Yes | Yes |  |
| Green | *Eubacterium eligens* | 18 | 0.13 | 0.01 | 0.94 | 0.63 |  |  |  |
| Green | *Intestinimonas butyriciproducens* | 16 | 0.12 | 0.02 | 0.94 | 0.44 |  |  |  |
| Green | *Faecalibacterium prausnitzii* | 16 | 0.12 | 0.03 | 0.92 | 0.55 |  |  |  |
| Green | *Eubacterium rectale* | 16 | 0.12 | 0.00 | 0.89 | 0.57 |  |  |  |
| Green | *Coprococcus comes* | 15 | 0.11 | 0.01 | 0.90 | 0.51 |  |  |  |
| Green | *Bifidobacterium longum* | 14 | 0.10 | 0.02 | 0.92 | 0.40 |  |  |  |
| Green | *Fusicatenibacter saccharivorans* | 13 | 0.10 | 0.00 | 0.89 | 0.63 |  |  |  |
| Green | *Gemmiger formicilis* | 12 | 0.09 | 0.02 | 0.85 | 0.42 |  |  |  |
| Green | *Eubacterium ramulus* | 12 | 0.09 | 0.00 | 0.83 | 0.43 |  |  |  |
| Green | *Roseburia hominis* | 11 | 0.08 | 0.00 | 0.84 | 0.37 |  |  |  |
| Green | *Agathobaculum butyriciproducens* | 7 | 0.05 | 0.00 | 0.79 | 0.31 |  |  |  |
| Green | *Adlercreutzia equolifaciens* | 7 | 0.05 | 0.00 | 0.82 | 0.23 |  |  |  |
| Green | *Asaccharobacter celatus* | 7 | 0.05 | 0.00 | 0.82 | 0.23 |  |  |  |
| Green | *Bifidobacterium adolescentis* | 7 | 0.05 | 0.00 | 0.78 | 0.27 |  |  |  |
| Green | *Bilophila wadsworthia* | 6 | 0.04 | 0.01 | 0.78 | 0.22 |  |  |  |
| Green | *Roseburia faecis* | 6 | 0.04 | 0.00 | 0.74 | 0.18 |  |  |  |
| Green | *Holdemanella biformis* | 6 | 0.04 | 0.00 | 0.77 | 0.21 |  |  |  |
| Green | *Roseburia intestinalis* | 5 | 0.04 | 0.00 | 0.71 | 0.10 |  |  |  |
| Green | *Escherichia coli* | 2 | 0.01 | 0.00 | 0.69 | 0.09 |  |  |  |
| Green | *Prevotella copri* | 1 | 0.01 | 0.00 | 0.57 | 0.02 |  |  |  |
| Light blue | *Odoribacter splanchnicus* | 15 | 0.11 | 0.08 | 0.92 | 0.23 | Yes | Yes |  |
| Light blue | *Alistipes indistinctus* | 14 | 0.10 | 0.09 | 0.90 | 0.14 |  |  |  |
| Light blue | *Oscillibacter sp CAG 241* | 10 | 0.07 | 0.03 | 0.85 | 0.22 |  |  |  |
| Light blue | *Eubacterium siraeum* | 9 | 0.07 | 0.01 | 0.84 | 0.19 |  |  |  |
| Light blue | *Parabacteroides merdae* | 7 | 0.05 | 0.02 | 0.78 | 0.10 |  |  |  |
| Light blue | *Butyricimonas virosa* | 7 | 0.05 | 0.04 | 0.78 | 0.06 |  |  |  |
| Light blue | *Alistipes putredinis* | 7 | 0.05 | 0.01 | 0.70 | 0.03 |  |  |  |
| Light blue | *Barnesiella intestinihominis* | 7 | 0.05 | 0.03 | 0.75 | 0.03 |  |  |  |
| Light blue | *Alistipes finegoldii* | 6 | 0.04 | 0.03 | 0.74 | 0.05 |  |  |  |
| Light blue | *Bacteroides caccae* | 6 | 0.04 | 0.02 | 0.70 | 0.02 |  |  |  |
| Light blue | *Bacteroides dorei* | 4 | 0.03 | 0.00 | 0.65 | 0.01 |  |  |  |
| Light blue | *Methanobrevibacter smithii* | 4 | 0.03 | 0.01 | 0.68 | 0.02 |  |  |  |
| Light blue | *Bacteroides vulgatus* | 4 | 0.03 | 0.00 | 0.61 | 0.01 |  |  |  |
| Light blue | *Bacteroides xylanisolvens* | 4 | 0.03 | 0.00 | 0.66 | 0.02 |  |  |  |
| Light blue | *Bacteroides nordii* | 4 | 0.03 | 0.02 | 0.68 | 0.02 |  |  |  |
| Light blue | *Parabacteroides distasonis* | 4 | 0.03 | 0.00 | 0.63 | 0.02 |  |  |  |
| Light blue | *Eubacterium limosum* | 4 | 0.03 | 0.02 | 0.68 | 0.01 |  |  |  |
| Light blue | *Butyricimonas synergistica* | 4 | 0.03 | 0.04 | 0.65 | 0.01 |  |  |  |
| Light blue | *Phascolarctobacterium faecium* | 3 | 0.02 | 0.02 | 0.56 | 0.00 |  |  |  |
| Light blue | *Parabacteroides goldsteinii* | 3 | 0.02 | 0.00 | 0.69 | 0.04 |  |  |  |
| Light blue | *Bacteroides finegoldii* | 3 | 0.02 | 0.02 | 0.51 | 0.00 |  |  |  |
| Light blue | *Christensenella minuta* | 3 | 0.02 | 0.00 | 0.63 | 0.01 |  |  |  |
| Light blue | *Clostridium sp CAG 58* | 3 | 0.02 | 0.01 | 0.69 | 0.08 |  |  |  |
| Light blue | *Bacteroides massiliensis* | 2 | 0.01 | 0.01 | 0.55 | 0.00 |  |  |  |
| Light blue | *Holdemania filiformis* | 1 | 0.01 | 0.00 | 0.43 | 0.00 |  |  |  |
| Light blue | *Bacteroides stercoris* | 1 | 0.01 | 0.00 | 0.50 | 0.00 |  |  |  |
| Light blue | *Paraprevotella xylaniphila* | 1 | 0.01 | 0.00 | 0.40 | 0.00 |  |  |  |
| Light blue | *Veillonella parvula* | 1 | 0.01 | 0.00 | 0.49 | 0.00 |  |  |  |
| Steel blue | *Firmicutes bacterium CAG 83* | 8 | 0.06 | 0.03 | 0.84 | 0.16 |  |  |  |
| Steel blue | *Faecalicatena orotica* | 3 | 0.02 | 0.00 | 0.62 | 0.01 |  |  |  |
| Steel blue | *Clostridium hylemonae* | 2 | 0.01 | 0.00 | 0.58 | 0.01 |  |  |  |
| Purple | *Gordonibacter pamelaeae* | 21 | 0.15 | 0.08 | 1.01 | 0.32 | Yes | Yes |  |
| Purple | *Flavonifractor plautii* | 13 | 0.10 | 0.04 | 0.86 | 0.08 |  |  |  |
| Purple | *Clostridium leptum* | 12 | 0.09 | 0.03 | 0.92 | 0.18 |  |  |  |
| Purple | *Ruminococcus gnavus* | 12 | 0.09 | 0.03 | 0.88 | 0.18 |  |  |  |
| Purple | *Clostridium scindens* | 12 | 0.09 | 0.04 | 0.85 | 0.07 |  |  |  |
| Purple | *Eggerthella lenta* | 11 | 0.08 | 0.03 | 0.92 | 0.13 |  |  |  |
| Purple | *Clostridium lavalense* | 11 | 0.08 | 0.06 | 0.85 | 0.06 |  |  |  |
| Purple | *Eisenbergiella tayi* | 10 | 0.07 | 0.01 | 0.85 | 0.17 |  |  |  |
| Purple | *Erysipelatoclostridium ramosum* | 9 | 0.07 | 0.01 | 0.77 | 0.04 |  |  |  |
| Purple | *Clostridium bolteae* | 9 | 0.07 | 0.04 | 0.80 | 0.03 |  |  |  |
| Purple | *Ruthenibacterium lactatiformans* | 8 | 0.06 | 0.00 | 0.78 | 0.06 |  |  |  |
| Purple | *Lawsonibacter asaccharolyticus* | 7 | 0.05 | 0.01 | 0.79 | 0.07 |  |  |  |
| Purple | *Blautia producta* | 7 | 0.05 | 0.00 | 0.73 | 0.04 |  |  |  |
| Purple | *Bacteroides uniformis* | 7 | 0.05 | 0.02 | 0.74 | 0.02 |  |  |  |
| Purple | *Hungatella hathewayi* | 6 | 0.04 | 0.01 | 0.74 | 0.04 |  |  |  |
| Purple | *Blautia coccoides* | 6 | 0.04 | 0.01 | 0.73 | 0.02 |  |  |  |
| Purple | *Catabacter hongkongensis* | 6 | 0.04 | 0.01 | 0.70 | 0.01 |  |  |  |
| Purple | *Clostridium citroniae* | 5 | 0.04 | 0.00 | 0.67 | 0.01 |  |  |  |
| Purple | *Parasutterella excrementihominis* | 5 | 0.04 | 0.01 | 0.72 | 0.01 |  |  |  |
| Purple | *Clostridium innocuum* | 5 | 0.04 | 0.00 | 0.71 | 0.04 |  |  |  |
| Purple | *Clostridium spiroforme* | 4 | 0.03 | 0.01 | 0.70 | 0.05 |  |  |  |
| Purple | *Streptococcus thermophilus* | 4 | 0.03 | 0.01 | 0.68 | 0.02 |  |  |  |
| Purple | *Clostridium symbiosum* | 4 | 0.03 | 0.00 | 0.70 | 0.02 |  |  |  |
| Purple | *Turicimonas muris* | 4 | 0.03 | 0.00 | 0.69 | 0.01 |  |  |  |
| Purple | *Blautia hydrogenotrophica* | 3 | 0.02 | 0.00 | 0.68 | 0.04 |  |  |  |
| Purple | *Bacteroides ovatus* | 3 | 0.02 | 0.00 | 0.72 | 0.06 |  |  |  |
| Purple | *Firmicutes bacterium CAG 145* | 3 | 0.02 | 0.00 | 0.66 | 0.02 |  |  |  |
| Purple | *Proteobacteria bacterium CAG 139* | 3 | 0.02 | 0.00 | 0.67 | 0.01 |  |  |  |
| Purple | *Clostridium bolteae CAG 59* | 3 | 0.02 | 0.00 | 0.62 | 0.00 |  |  |  |
| Purple | *Sellimonas intestinalis* | 2 | 0.01 | 0.00 | 0.65 | 0.01 |  |  |  |
| Pink | *Lactobacillus paragasseri* | 3 | 0.02 | 0.02 | 0.67 | 0.05 |  |  |  |
| Pink | *Lactobacillus fermentum* | 3 | 0.02 | 0.00 | 0.67 | 0.05 |  |  |  |
| Pink | *Lactobacillus gasseri* | 2 | 0.01 | 0.00 | 0.53 | 0.01 |  |  |  |
| Light grey | *Enterococcus faecalis* | NA | 0.00 | 0.00 | 0.00 | 0.00 |  |  |  |
| Light grey | *Akkermansia muciniphila* | NA | 0.00 | 0.00 | 0.00 | 0.00 |  |  |  |
| Light grey | *Dielma fastidiosa* | NA | 0.00 | 0.00 | 0.00 | 0.00 |  |  |  |
| Light grey | *Enterococcus faecium* | NA | 0.00 | 0.00 | 0.00 | 0.00 |  |  |  |
| Light grey | *Harryflintia acetispora* | NA | 0.00 | 0.00 | 0.00 | 0.00 |  |  |  |
| Light grey | *Pseudomonas aeruginosa group* | NA | 0.00 | 0.00 | 0.00 | 0.00 |  |  | Yes |
| Light grey | *Clostridium clostridioforme* | NA | 0.00 | 0.00 | 0.00 | 0.00 |  |  |  |
| Light grey | *Bacteroides fragilis* | NA | 0.00 | 0.00 | 0.00 | 0.00 |  |  |  |
| Light grey | *Bacteroides salyersiae* | NA | 0.00 | 0.00 | 0.00 | 0.00 |  |  |  |
| Light grey | *Lactobacillus rhamnosus* | NA | 0.00 | 0.00 | 0.00 | 0.00 |  |  |  |
| Light grey | *Anaerotignum lactatifermentans* | NA | 0.00 | 0.00 | 0.00 | 0.00 |  |  |  |
| Light grey | *Bacteroides thetaiotaomicron* | NA | 0.00 | 0.00 | 0.00 | 0.00 |  |  |  |
| Light grey | *Eubacterium callanderi* | NA | 0.00 | 0.00 | 0.00 | 0.00 |  |  |  |
| Light grey | *Klebsiella pneumoniae* | NA | 0.00 | 0.00 | 0.00 | 0.00 |  |  |  |
| Light grey | *Coprobacillus cateniformis* | NA | 0.00 | 0.00 | 0.00 | 0.00 |  |  | Yes |
| Light grey | *Clostridium methylpentosum* | NA | 0.00 | 0.00 | 0.00 | 0.00 |  |  |  |
| Light grey | *Bifidobacterium breve* | NA | 0.00 | 0.00 | 0.00 | 0.00 |  |  |  |
| Light grey | *Anaerostipes caccae* | NA | 0.00 | 0.00 | 0.00 | 0.00 |  |  |  |
| Light grey | *Bacteroides cellulosilyticus* | NA | 0.00 | 0.00 | 0.00 | 0.00 |  |  |  |
| Light grey | *Acidaminococcus intestini* | NA | 0.00 | 0.00 | 0.00 | 0.00 |  |  | Yes |
| Light grey | *Candida albicans* | NA | 0.00 | 0.00 | 0.00 | 0.00 |  |  |  |

**Table S6. Multivariable linear modeling results (predicted stool metabolites)**

| **feature** | **metadata** | **value** | **coef** | **stderr** | **N** | **N.not.0** | **pval** | **qval** |
| --- | --- | --- | --- | --- | --- | --- | --- | --- |
| adrenic.acid | covid_severity | Severe | 0.78 | 0.11 | 241 | 241 | 2.01E-10 | 2.57E-07 |
| eicosatrienoic.acid | covid_severity | Severe | 0.70 | 0.10 | 241 | 241 | 6.81E-10 | 4.36E-07 |
| docosapentaenoic.acid | covid_severity | Severe | 0.38 | 0.06 | 241 | 241 | 6.32E-09 | 2.70E-06 |
| arachidonic.acid | covid_severity | Severe | 0.37 | 0.06 | 241 | 241 | 4.97E-08 | 1.16E-05 |
| C18.0.SM | covid_severity | Severe | 0.29 | 0.05 | 241 | 241 | 5.73E-08 | 1.16E-05 |
| thymine | covid_severity | Severe | -0.21 | 0.04 | 241 | 241 | 6.12E-08 | 1.16E-05 |
| bilirubin | covid_severity | Severe | 0.26 | 0.05 | 241 | 241 | 6.32E-08 | 1.16E-05 |
| palmitoyl.glycerol | covid_severity | Severe | 0.24 | 0.04 | 241 | 241 | 7.46E-08 | 1.19E-05 |
| C18.1.CE | covid_severity | Severe | 0.31 | 0.05 | 241 | 241 | 8.63E-08 | 1.22E-05 |
| docosapentaenoate | covid_severity | Severe | 0.29 | 0.05 | 241 | 241 | 9.54E-08 | 1.22E-05 |
| docosahexaenoic.acid | covid_severity | Severe | 0.21 | 0.04 | 241 | 241 | 1.27E-07 | 1.48E-05 |
| dimethyllysine | covid_severity | Severe | 0.26 | 0.05 | 241 | 241 | 2.83E-07 | 3.02E-05 |
| undecanedionate | covid_severity | Severe | -0.49 | 0.09 | 241 | 241 | 3.28E-07 | 3.23E-05 |
| C16.0.LPC | covid_severity | Severe | 0.53 | 0.10 | 241 | 241 | 3.91E-07 | 3.33E-05 |
| erythronic.acid | covid_severity | Severe | 0.21 | 0.04 | 241 | 241 | 3.84E-07 | 3.33E-05 |
| malonate | covid_severity | Severe | 0.24 | 0.05 | 241 | 241 | 5.59E-07 | 4.21E-05 |
| ADMA | covid_severity | Severe | 0.53 | 0.10 | 241 | 241 | 5.30E-07 | 4.21E-05 |
| C16.0.ceramide..d18.1. | covid_severity | Severe | 0.26 | 0.05 | 241 | 241 | 1.16E-06 | 7.79E-05 |
| pantothenate | covid_severity | Severe | 0.14 | 0.03 | 241 | 241 | 1.15E-06 | 7.79E-05 |
| C18.0e.MAG | covid_severity | Severe | 0.22 | 0.04 | 241 | 241 | 1.29E-06 | 8.27E-05 |
| pyridoxamine | covid_severity | Severe | -0.30 | 0.06 | 241 | 241 | 1.47E-06 | 8.97E-05 |
| diacetylspermine | covid_severity | Severe | 0.38 | 0.08 | 241 | 241 | 1.95E-06 | 0.000113243 |
| imidazole.propionate | covid_severity | Severe | 0.46 | 0.09 | 241 | 241 | 2.45E-06 | 0.000136517 |
| xanthine | read_depth | read_depth | -0.07 | 0.01 | 241 | 241 | 2.91E-06 | 0.000155296 |
| X2.hydroxyphenethylamine | covid_severity | Severe | 0.26 | 0.06 | 241 | 241 | 3.86E-06 | 0.00019768 |
| azelate | covid_severity | Severe | -0.21 | 0.04 | 241 | 241 | 4.14E-06 | 0.000204031 |
| C2.carnitine | covid_severity | Severe | 0.98 | 0.21 | 241 | 241 | 5.05E-06 | 0.000239389 |
| N.acetylputrescine | covid_severity | Severe | 0.81 | 0.17 | 241 | 241 | 6.67E-06 | 0.000301382 |
| creatine | covid_severity | Severe | 0.61 | 0.13 | 241 | 241 | 6.83E-06 | 0.000301382 |
| cholate | covid_severity | Severe | 1.00 | 0.22 | 241 | 241 | 8.32E-06 | 0.000354993 |
| caproic.acid | covid_severity | Severe | -0.22 | 0.05 | 241 | 241 | 8.62E-06 | 0.000355995 |
| glutamate | read_depth | read_depth | -0.03 | 0.01 | 241 | 241 | 9.44E-06 | 0.000366065 |
| C16.carnitine | covid_severity | Severe | 0.25 | 0.05 | 241 | 241 | 9.40E-06 | 0.000366065 |
| fructose.glucose.galactose. | covid_severity | Severe | 0.13 | 0.03 | 241 | 241 | 1.03E-05 | 0.000385302 |
| trimethyllysine | covid_severity | Severe | 0.17 | 0.04 | 241 | 241 | 1.05E-05 | 0.000385302 |
| inosine | time_from_admit | time_from_admit | 0.00 | 0.00 | 241 | 241 | 1.40E-05 | 0.000496023 |
| nicotinic.acid | time_from_admit | time_from_admit | 0.00 | 0.00 | 241 | 241 | 1.52E-05 | 0.000527136 |
| chenodeoxycholate | covid_severity | Severe | 0.83 | 0.19 | 241 | 241 | 1.64E-05 | 0.000550792 |
| ADMA.SDMA. | covid_severity | Severe | 0.34 | 0.08 | 241 | 241 | 1.97E-05 | 0.000646026 |
| hypoxanthine | covid_severity | Severe | 0.13 | 0.03 | 241 | 241 | 2.24E-05 | 0.000717207 |
| citrulline | read_depth | read_depth | -0.16 | 0.04 | 241 | 241 | 3.33E-05 | 0.001040072 |
| phytosphingosine | covid_severity | Severe | 0.29 | 0.07 | 241 | 241 | 4.77E-05 | 0.001453415 |
| deoxycholic.acid | read_depth | read_depth | 0.08 | 0.02 | 241 | 241 | 5.39E-05 | 0.00160493 |
| X2.hydroxymyristic.acid | covid_severity | Severe | -0.17 | 0.04 | 241 | 241 | 5.67E-05 | 0.00165003 |
| X7.methylguanine | read_depth | read_depth | -0.07 | 0.02 | 241 | 241 | 6.34E-05 | 0.001803744 |
| fructose.glucose.galactose. | read_depth | read_depth | -0.04 | 0.01 | 241 | 241 | 7.56E-05 | 0.002057552 |
| X3.methylxanthine | read_depth | read_depth | -0.07 | 0.02 | 241 | 241 | 7.50E-05 | 0.002057552 |
| X3.methyladipate.pimelate | covid_severity | Severe | -0.81 | 0.20 | 241 | 241 | 8.14E-05 | 0.002171103 |
| trimethyllysine | read_depth | read_depth | -0.05 | 0.01 | 241 | 241 | 8.37E-05 | 0.002185978 |
| ketodeoxycholate | covid_severity | Severe | 0.62 | 0.16 | 241 | 241 | 0.00014028 | 0.003591179 |
| N.acetylspermidine | read_depth | read_depth | -0.04 | 0.01 | 241 | 241 | 0.000144171 | 0.003618403 |
| hypoxanthine | read_depth | read_depth | -0.04 | 0.01 | 241 | 241 | 0.000154438 | 0.003729819 |
| lithocholate | read_depth | read_depth | 0.14 | 0.04 | 241 | 241 | 0.000152802 | 0.003729819 |
| lithocholic.acid | covid_severity | Severe | -0.36 | 0.09 | 241 | 241 | 0.000162452 | 0.003780703 |
| urobilin | read_depth | read_depth | 0.16 | 0.04 | 241 | 241 | 0.000160045 | 0.003780703 |
| malonate | read_depth | read_depth | -0.06 | 0.02 | 241 | 241 | 0.000205 | 0.004648292 |
| N.acetylhistidine | covid_severity | Severe | 0.10 | 0.03 | 241 | 241 | 0.000206994 | 0.004648292 |
| propionate | time_from_admit | time_from_admit | 0.00 | 0.00 | 241 | 241 | 0.000220761 | 0.004871975 |
| ADMA.SDMA. | read_depth | read_depth | -0.10 | 0.03 | 241 | 241 | 0.000224843 | 0.004877953 |
| nicotinate | time_from_admit | time_from_admit | 0.00 | 0.00 | 241 | 241 | 0.00023257 | 0.0049615 |
| pantothenate | read_depth | read_depth | -0.04 | 0.01 | 241 | 241 | 0.00023697 | 0.004972487 |
| deoxyinosine | covid_severity | Severe | -0.10 | 0.03 | 241 | 241 | 0.000241061 | 0.004976737 |
| C16.0.ceramide..d18.1. | read_depth | read_depth | -0.07 | 0.02 | 241 | 241 | 0.000294651 | 0.005893015 |
| dimethyllysine | read_depth | read_depth | -0.06 | 0.02 | 241 | 241 | 0.000293893 | 0.005893015 |
| pyridoxamine | time_from_admit | time_from_admit | -0.01 | 0.00 | 241 | 241 | 0.000308764 | 0.006080272 |
| threosphingosine | covid_severity | Severe | 0.22 | 0.06 | 241 | 241 | 0.000323812 | 0.006279998 |
| C18.0.SM | read_depth | read_depth | -0.06 | 0.02 | 241 | 241 | 0.000393055 | 0.007509101 |
| butyrate...isobutytare. | time_from_admit | time_from_admit | 0.00 | 0.00 | 241 | 241 | 0.000413187 | 0.007777632 |
| N.acetylhistidine | read_depth | read_depth | -0.03 | 0.01 | 241 | 241 | 0.00044346 | 0.008226504 |
| palmitoyl.glycerol | read_depth | read_depth | -0.05 | 0.01 | 241 | 241 | 0.000485578 | 0.008681929 |
| C18.0e.MAG | read_depth | read_depth | -0.05 | 0.02 | 241 | 241 | 0.000495141 | 0.008681929 |
| putrescine | covid_severity | Severe | 0.17 | 0.05 | 241 | 241 | 0.000483709 | 0.008681929 |
| urobilin. | covid_severity | Severe | -0.34 | 0.10 | 241 | 241 | 0.000488661 | 0.008681929 |
| docosahexaenoic.acid | read_depth | read_depth | -0.05 | 0.01 | 241 | 241 | 0.000514905 | 0.008906466 |
| threosphingosine | read_depth | read_depth | -0.08 | 0.02 | 241 | 241 | 0.000529861 | 0.009042959 |
| C18.1.CE | read_depth | read_depth | -0.07 | 0.02 | 241 | 241 | 0.00054283 | 0.0091424 |
| urobilin | covid_severity | Severe | -0.44 | 0.12 | 241 | 241 | 0.000577728 | 0.009603783 |
| lithocholic.acid | read_depth | read_depth | 0.11 | 0.03 | 241 | 241 | 0.000586122 | 0.009618409 |
| N.acetylglutamic.acid | time_from_admit | time_from_admit | 0.00 | 0.00 | 241 | 241 | 0.000620659 | 0.010056251 |
| uracil | read_depth | read_depth | -0.03 | 0.01 | 241 | 241 | 0.000658101 | 0.010529618 |
| thymine | on_abx | TRUE | -0.12 | 0.03 | 241 | 241 | 0.000673312 | 0.010639998 |
| uracil | ethnicity | Hispanic | 0.11 | 0.03 | 241 | 241 | 0.000693054 | 0.010787418 |
| N.acetylglutamate | time_from_admit | time_from_admit | 0.00 | 0.00 | 241 | 241 | 0.000699497 | 0.010787418 |
| X3.methyladipate.pimelate | read_depth | read_depth | 0.24 | 0.07 | 241 | 241 | 0.000754994 | 0.011504669 |
| X7.methylguanine | covid_severity | Severe | 0.17 | 0.05 | 241 | 241 | 0.000812039 | 0.01222835 |
| thymine | time_from_admit | time_from_admit | 0.00 | 0.00 | 241 | 241 | 0.000831015 | 0.012368592 |
| N.acetylglutamic.acid | covid_severity | Severe | -0.14 | 0.04 | 241 | 241 | 0.000858091 | 0.012624793 |
| N.acetylputrescine | read_depth | read_depth | -0.19 | 0.06 | 241 | 241 | 0.00089407 | 0.013004654 |
| alpha.muricholate | covid_severity | Severe | 0.47 | 0.14 | 241 | 241 | 0.000930485 | 0.013233568 |
| nicotinic.acid | covid_severity | Severe | -0.15 | 0.04 | 241 | 241 | 0.00092435 | 0.013233568 |
| X3.methyladipate.pimelate | time_from_admit | time_from_admit | -0.02 | 0.00 | 241 | 241 | 0.000960216 | 0.013506333 |
| chenodeoxycholate.deoxycholate. | read_depth | read_depth | 0.06 | 0.02 | 241 | 241 | 0.000992582 | 0.013809831 |
| erythronic.acid | read_depth | read_depth | -0.05 | 0.01 | 241 | 241 | 0.001095172 | 0.014756004 |
| putrescine | read_depth | read_depth | -0.05 | 0.02 | 241 | 241 | 0.001084304 | 0.014756004 |
| X2.hydroxyphenethylamine | read_depth | read_depth | -0.06 | 0.02 | 241 | 241 | 0.001078365 | 0.014756004 |
| urobilin. | read_depth | read_depth | 0.11 | 0.03 | 241 | 241 | 0.001120246 | 0.014936615 |
| glucurote | read_depth | read_depth | -0.03 | 0.01 | 241 | 241 | 0.001197202 | 0.015798128 |
| imidazole.propionate | time_from_admit | time_from_admit | 0.01 | 0.00 | 241 | 241 | 0.001243777 | 0.01624525 |
| arachidonic.acid | read_depth | read_depth | -0.07 | 0.02 | 241 | 241 | 0.001262994 | 0.016329623 |
| glutamate | ethnicity | Hispanic | 0.07 | 0.02 | 241 | 241 | 0.001626736 | 0.020822219 |
| adrenic.acid | read_depth | read_depth | -0.12 | 0.04 | 241 | 241 | 0.001948452 | 0.024693254 |
| N.acetylglutamic.acid | read_depth | read_depth | 0.04 | 0.01 | 241 | 241 | 0.002040459 | 0.025605757 |
| diacetylspermine | read_depth | read_depth | -0.08 | 0.03 | 241 | 241 | 0.002086323 | 0.025927126 |
| bilirubin | read_depth | read_depth | -0.05 | 0.02 | 241 | 241 | 0.002230297 | 0.027449812 |
| chenodeoxycholate | read_depth | read_depth | -0.18 | 0.06 | 241 | 241 | 0.002289898 | 0.027914943 |
| deoxycholic.acid | covid_severity | Severe | -0.17 | 0.05 | 241 | 241 | 0.002443409 | 0.029505315 |
| C16.0.LPC | read_depth | read_depth | -0.10 | 0.03 | 241 | 241 | 0.002598049 | 0.03107947 |
| methylbutyric.aid...valeric...isovaleric. | ethnicity | Hispanic | 0.09 | 0.03 | 241 | 241 | 0.002827162 | 0.033507111 |
| phenylacetate | ethnicity | Hispanic | 0.10 | 0.03 | 241 | 241 | 0.002939101 | 0.034200445 |
| docosapentaenoate | read_depth | read_depth | -0.05 | 0.02 | 241 | 241 | 0.002938817 | 0.034200445 |
| propionate | covid_severity | Severe | -0.12 | 0.04 | 241 | 241 | 0.002966524 | 0.034208565 |
| butyrate...isobutytare. | covid_severity | Severe | -0.10 | 0.03 | 241 | 241 | 0.003021168 | 0.034222084 |
| lithocholate | covid_severity | Severe | -0.31 | 0.10 | 241 | 241 | 0.003011726 | 0.034222084 |
| X2.hydroxymyristic.acid | time_from_admit | time_from_admit | 0.00 | 0.00 | 241 | 241 | 0.003241801 | 0.036399171 |
| taurine | time_from_admit | time_from_admit | 0.00 | 0.00 | 241 | 241 | 0.00340227 | 0.03786874 |
| N.acetylglutamate | covid_severity | Severe | -0.11 | 0.04 | 241 | 241 | 0.003846444 | 0.042443522 |
| phenylacetate | time_from_admit | time_from_admit | 0.00 | 0.00 | 241 | 241 | 0.004067184 | 0.044495684 |
| phenylacetate | read_depth | read_depth | -0.03 | 0.01 | 241 | 241 | 0.004389526 | 0.047571982 |
| N.acetylputrescine | time_from_admit | time_from_admit | 0.01 | 0.00 | 241 | 241 | 0.004422708 | 0.047571982 |
| nicotinic.acid | tx_remdesivir | 1 | 0.15 | 0.05 | 241 | 241 | 0.004483081 | 0.047819532 |
| hydrocinnamic.acid | read_depth | read_depth | 0.12 | 0.04 | 241 | 241 | 0.004603039 | 0.048693304 |
| chenodeoxycholate | time_from_admit | time_from_admit | 0.01 | 0.00 | 241 | 241 | 0.005141217 | 0.053502095 |
| azelaic.acid | covid_severity | Severe | -0.06 | 0.02 | 241 | 241 | 0.005138647 | 0.053502095 |
| creatine | read_depth | read_depth | -0.12 | 0.04 | 241 | 241 | 0.005252932 | 0.05422381 |
| sebacate | tx_remdesivir | 1 | 0.11 | 0.04 | 241 | 241 | 0.005381804 | 0.055109672 |
| citrulline | time_from_admit | time_from_admit | -0.01 | 0.00 | 241 | 241 | 0.005575493 | 0.056639925 |
| cholate | read_depth | read_depth | -0.19 | 0.07 | 241 | 241 | 0.005819732 | 0.058655568 |
| docosapentaenoic.acid | read_depth | read_depth | -0.06 | 0.02 | 241 | 241 | 0.005970239 | 0.059354859 |
| uracil | on_abx | TRUE | -0.07 | 0.03 | 241 | 241 | 0.006010206 | 0.059354859 |
| inosine | covid_severity | Severe | 0.10 | 0.04 | 241 | 241 | 0.006028228 | 0.059354859 |
| bilirubin | time_from_admit | time_from_admit | 0.00 | 0.00 | 241 | 241 | 0.006748826 | 0.065942732 |
| methylbutyric.aid...valeric...isovaleric. | race | Other | -0.12 | 0.04 | 241 | 241 | 0.00690185 | 0.066780861 |
| citrulline | race | Mixed | 0.56 | 0.20 | 241 | 241 | 0.006938949 | 0.066780861 |
| N.acetylhistidine | ethnicity | Hispanic | 0.08 | 0.03 | 241 | 241 | 0.007012465 | 0.066984738 |
| phytosphingosine | read_depth | read_depth | -0.06 | 0.02 | 241 | 241 | 0.007402214 | 0.070183951 |
| xanthine | covid_severity | Severe | 0.10 | 0.04 | 241 | 241 | 0.008634912 | 0.081269758 |
| X3.methylxanthine | covid_severity | Severe | 0.13 | 0.05 | 241 | 241 | 0.008781837 | 0.082049277 |
| cytosine | covid_severity | Severe | -0.20 | 0.08 | 241 | 241 | 0.009102144 | 0.084425683 |
| cholesterol | read_depth | read_depth | -0.06 | 0.02 | 241 | 241 | 0.009435542 | 0.086888447 |
| pseudouridine | ethnicity | Hispanic | 0.06 | 0.02 | 241 | 241 | 0.009826644 | 0.089843606 |
| C16.carnitine | read_depth | read_depth | -0.05 | 0.02 | 241 | 241 | 0.010585145 | 0.096092096 |
| hypoxanthine | ethnicity | Hispanic | 0.08 | 0.03 | 241 | 241 | 0.011708437 | 0.105540844 |
| sebacate | covid_severity | Severe | -0.09 | 0.03 | 241 | 241 | 0.012193619 | 0.109145682 |
| cholesterol | race | Asian | 0.35 | 0.14 | 241 | 241 | 0.012816658 | 0.113925845 |
| phenylacetate | race | Other | -0.11 | 0.04 | 241 | 241 | 0.013178704 | 0.116336142 |
| butyrate...isobutytare. | race | Other | -0.13 | 0.05 | 241 | 241 | 0.014286727 | 0.122744707 |
| N.acetylglutamate | read_depth | read_depth | 0.03 | 0.01 | 241 | 241 | 0.014288251 | 0.122744707 |
| X2.hydroxyphenethylamine | time_from_admit | time_from_admit | 0.00 | 0.00 | 241 | 241 | 0.014191965 | 0.122744707 |
| ADMA | read_depth | read_depth | -0.09 | 0.04 | 241 | 241 | 0.014123795 | 0.122744707 |
| pyridoxamine | age | age | -0.07 | 0.03 | 241 | 241 | 0.014941382 | 0.127499792 |
| pantothenate | ethnicity | Hispanic | 0.08 | 0.03 | 241 | 241 | 0.015604519 | 0.13227672 |
| chenodeoxycholate.deoxycholate. | covid_severity | Severe | -0.11 | 0.04 | 241 | 241 | 0.015847332 | 0.133451221 |
| cholate | time_from_admit | time_from_admit | 0.01 | 0.00 | 241 | 241 | 0.016180878 | 0.135369435 |
| butyrate...isobutytare. | ethnicity | Hispanic | 0.09 | 0.04 | 241 | 241 | 0.016962439 | 0.140986502 |
| nicotinate | ethnicity | Hispanic | 0.05 | 0.02 | 241 | 241 | 0.017291782 | 0.142796649 |
| citrulline | covid_severity | Severe | 0.23 | 0.10 | 241 | 241 | 0.0174318 | 0.143030156 |
| deoxyinosine | tx_remdesivir | 1 | 0.07 | 0.03 | 241 | 241 | 0.018183261 | 0.147485032 |
| trimethyllysine | ethnicity | Hispanic | 0.10 | 0.04 | 241 | 241 | 0.018205184 | 0.147485032 |
| pseudouridine | time_from_admit | time_from_admit | 0.00 | 0.00 | 241 | 241 | 0.01834276 | 0.147664985 |
| stearoyl.ethanolamide | covid_severity | Severe | 0.12 | 0.05 | 241 | 241 | 0.018515443 | 0.148123541 |
| eicosatrienoic.acid | read_depth | read_depth | -0.08 | 0.03 | 241 | 241 | 0.018875582 | 0.150066742 |
| alpha.muricholate | read_depth | read_depth | -0.12 | 0.05 | 241 | 241 | 0.019746625 | 0.156022713 |
| undecanedionate | read_depth | read_depth | 0.08 | 0.03 | 241 | 241 | 0.020261516 | 0.158894727 |
| eicosatrienoic.acid | on_abx | TRUE | 0.23 | 0.10 | 241 | 241 | 0.020358387 | 0.158894727 |
| linoleoyl.ethanolamide | race | Asian | 0.29 | 0.12 | 241 | 241 | 0.021418726 | 0.164167481 |
| pseudouridine | on_abx | TRUE | -0.05 | 0.02 | 241 | 241 | 0.021333498 | 0.164167481 |
| urobilin. | time_from_admit | time_from_admit | 0.00 | 0.00 | 241 | 241 | 0.021348001 | 0.164167481 |
| N.acetylglutamic.acid | tx_remdesivir | 1 | 0.11 | 0.05 | 241 | 241 | 0.021731141 | 0.1655706 |
| fructose.glucose.galactose. | ethnicity | Hispanic | 0.07 | 0.03 | 241 | 241 | 0.022479526 | 0.170259131 |
| pseudouridine | read_depth | read_depth | -0.02 | 0.01 | 241 | 241 | 0.022631406 | 0.170401177 |
| docosahexaenoic.acid | ethnicity | Hispanic | 0.10 | 0.04 | 241 | 241 | 0.023573815 | 0.17543304 |
| phenylacetate | covid_severity | Severe | 0.07 | 0.03 | 241 | 241 | 0.023463317 | 0.17543304 |
| ketodeoxycholate | time_from_admit | time_from_admit | 0.01 | 0.00 | 241 | 241 | 0.023884267 | 0.176715965 |
| adrenic.acid | time_from_admit | time_from_admit | 0.01 | 0.00 | 241 | 241 | 0.024328867 | 0.177210196 |
| propionate | read_depth | read_depth | -0.03 | 0.01 | 241 | 241 | 0.024366402 | 0.177210196 |
| cytosine | ethnicity | Hispanic | 0.20 | 0.09 | 241 | 241 | 0.024147941 | 0.177210196 |
| C18.0e.MAG | time_from_admit | time_from_admit | 0.00 | 0.00 | 241 | 241 | 0.024953079 | 0.180451644 |
| ketodeoxycholate | age | age | 0.18 | 0.08 | 241 | 241 | 0.025103284 | 0.180517998 |
| glucurote | ethnicity | Hispanic | 0.06 | 0.03 | 241 | 241 | 0.02535437 | 0.181304992 |
| azelate | time_from_admit | time_from_admit | 0.00 | 0.00 | 241 | 241 | 0.026171745 | 0.186110184 |
| azelate | read_depth | read_depth | 0.03 | 0.02 | 241 | 241 | 0.026599697 | 0.187074794 |
| caproic.acid | read_depth | read_depth | 0.04 | 0.02 | 241 | 241 | 0.026535835 | 0.187074794 |
| deoxycholic.acid | ethnicity | Hispanic | -0.13 | 0.06 | 241 | 241 | 0.027002475 | 0.187843302 |
| imidazole.propionate | read_depth | read_depth | -0.07 | 0.03 | 241 | 241 | 0.026933653 | 0.187843302 |
| palmitoyl.glycerol | ethnicity | Hispanic | 0.11 | 0.05 | 241 | 241 | 0.02798757 | 0.193643729 |
| C18.0e.MAG | ethnicity | Hispanic | 0.11 | 0.05 | 241 | 241 | 0.028688779 | 0.197428159 |
| pyridoxamine | read_depth | read_depth | 0.05 | 0.02 | 241 | 241 | 0.028891782 | 0.197761933 |
| imidazole.propionate | on_abx | TRUE | 0.19 | 0.09 | 241 | 241 | 0.030439837 | 0.207249953 |
| urobilin | time_from_admit | time_from_admit | -0.01 | 0.00 | 241 | 241 | 0.030964308 | 0.209290399 |
| N.acetylglutamate | tx_remdesivir | 1 | 0.09 | 0.04 | 241 | 241 | 0.031066544 | 0.209290399 |
| glutamate | covid_severity | Severe | 0.04 | 0.02 | 241 | 241 | 0.031418353 | 0.210552311 |
| N.oleoylethanolamine | race | Asian | 0.44 | 0.20 | 241 | 241 | 0.031618631 | 0.210790876 |
| nicotinate | read_depth | read_depth | -0.02 | 0.01 | 241 | 241 | 0.032675318 | 0.216706772 |
| propionate | male | yes | -0.07 | 0.03 | 241 | 241 | 0.033058138 | 0.218115551 |
| threosphingosine | ethnicity | Hispanic | 0.14 | 0.07 | 241 | 241 | 0.035235592 | 0.231290042 |
| threosphingosine | time_from_admit | time_from_admit | 0.00 | 0.00 | 241 | 241 | 0.035545903 | 0.232136511 |
| docosapentaenoic.acid | time_from_admit | time_from_admit | 0.00 | 0.00 | 241 | 241 | 0.036463818 | 0.23427333 |
| malonate | ethnicity | Hispanic | 0.11 | 0.05 | 241 | 241 | 0.03656699 | 0.23427333 |
| uracil | time_from_admit | time_from_admit | 0.00 | 0.00 | 241 | 241 | 0.036439648 | 0.23427333 |
| cytosine | race | Other | -0.26 | 0.12 | 241 | 241 | 0.036605208 | 0.23427333 |
| hydrocinnamic.acid | covid_severity | Severe | -0.26 | 0.12 | 241 | 241 | 0.038445241 | 0.244825415 |
| undecanedionate | time_from_admit | time_from_admit | 0.00 | 0.00 | 241 | 241 | 0.039082056 | 0.247648675 |
| stearoyl.ethanolamide | race | Asian | 0.22 | 0.11 | 241 | 241 | 0.039685653 | 0.249257849 |
| erythronic.acid | ethnicity | Hispanic | 0.09 | 0.04 | 241 | 241 | 0.03972547 | 0.249257849 |
| Results with FDR-corrected p-value <0.25 shown. | | | | | | | | |

**Figure S1. Study enrollment diagram**

**
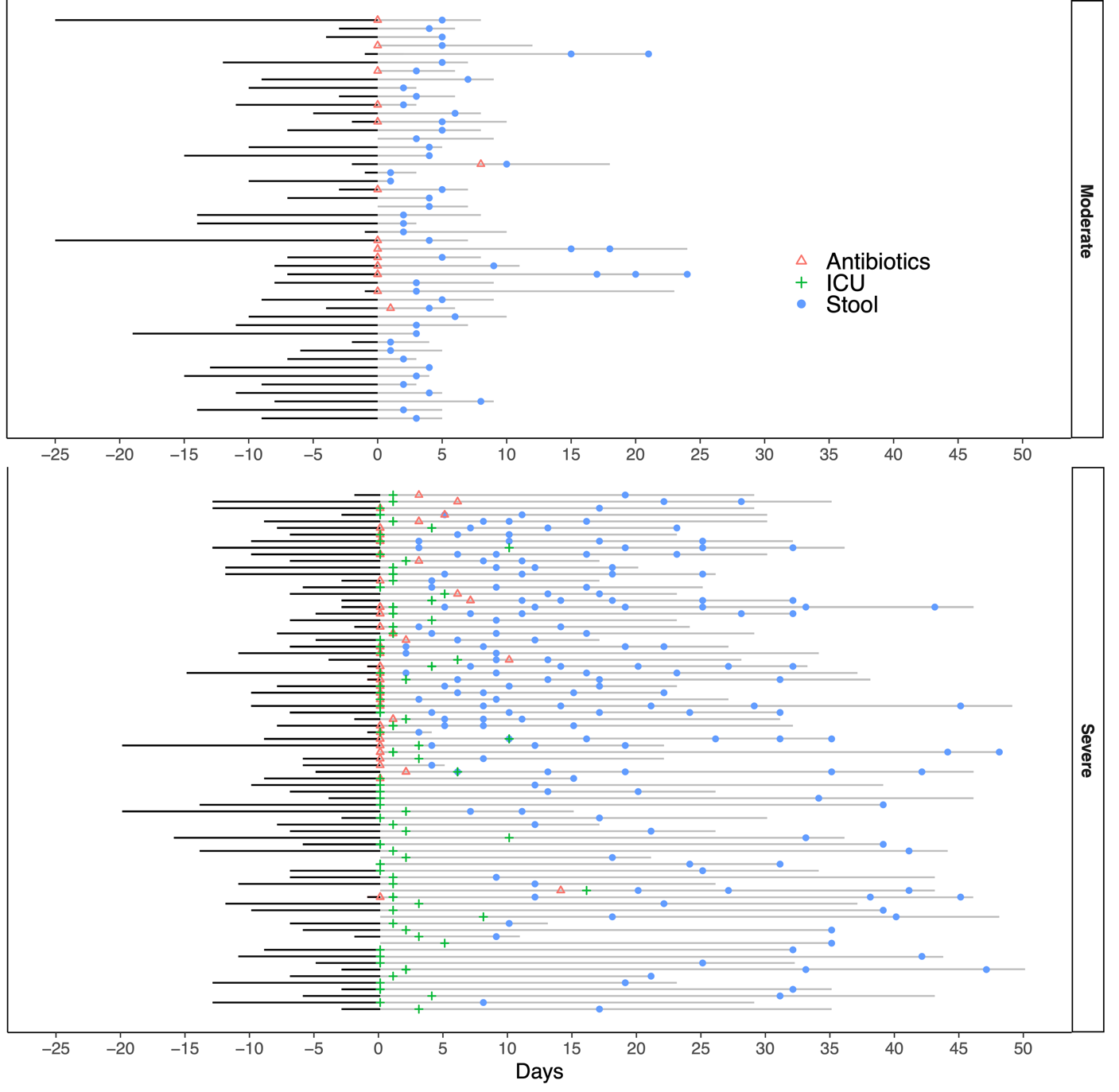
**

Sampling timeline with respect to onset of symptoms (black line) and hospital discharge/death (gray line) by participant. Timing of antibiotics (red triangle), admission to ICU (green cross), and stool sampling (blue circle) are denoted. Day 0 represents day of admission, and admissions longer than 50 days have been truncated for visualizations purposes.

**Figure S2. PCoA by batch**

**
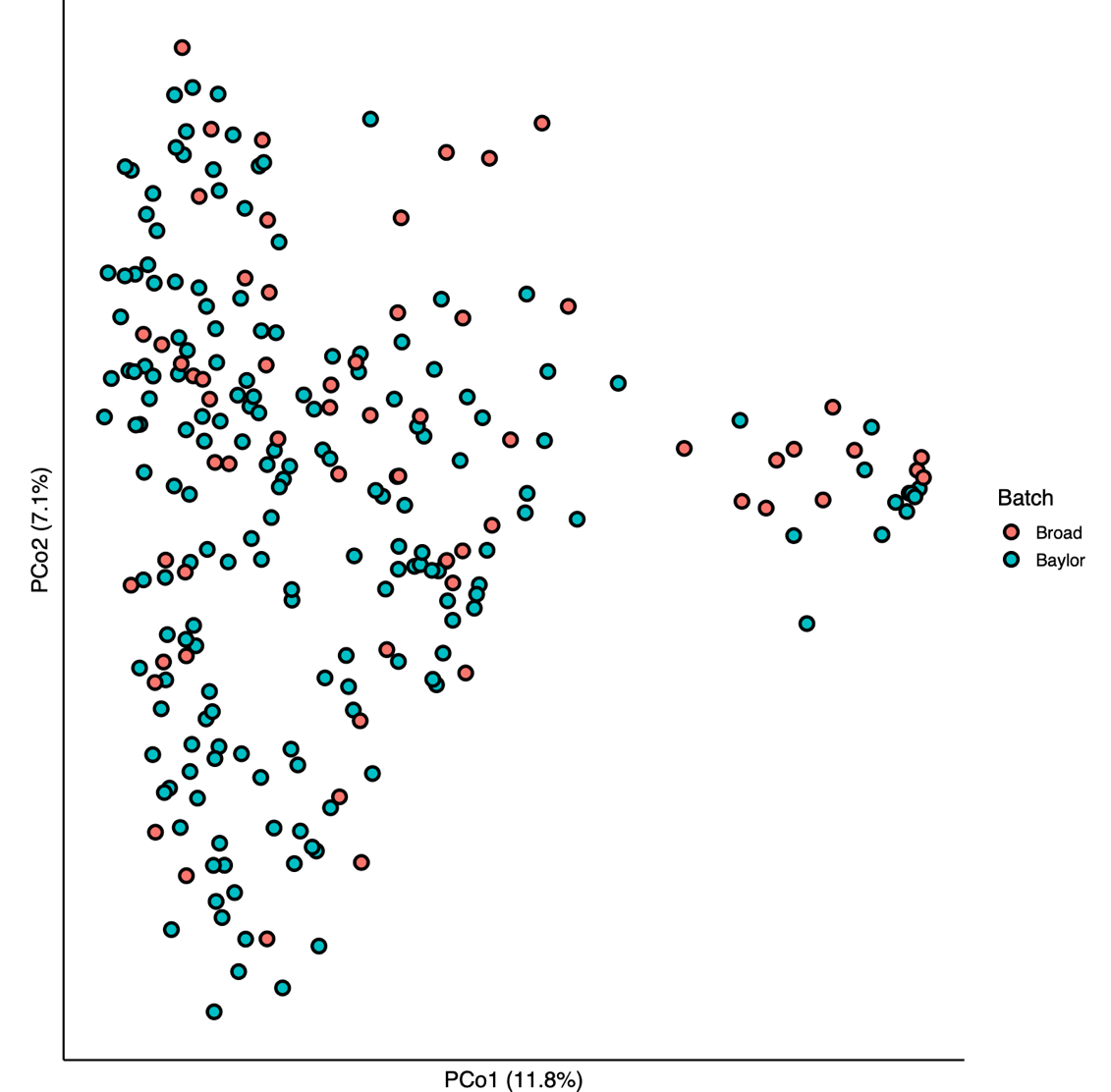
**

**Figure S3. Volcano plot for multivariable linear modeling results (MetaCyc pathways)**

**
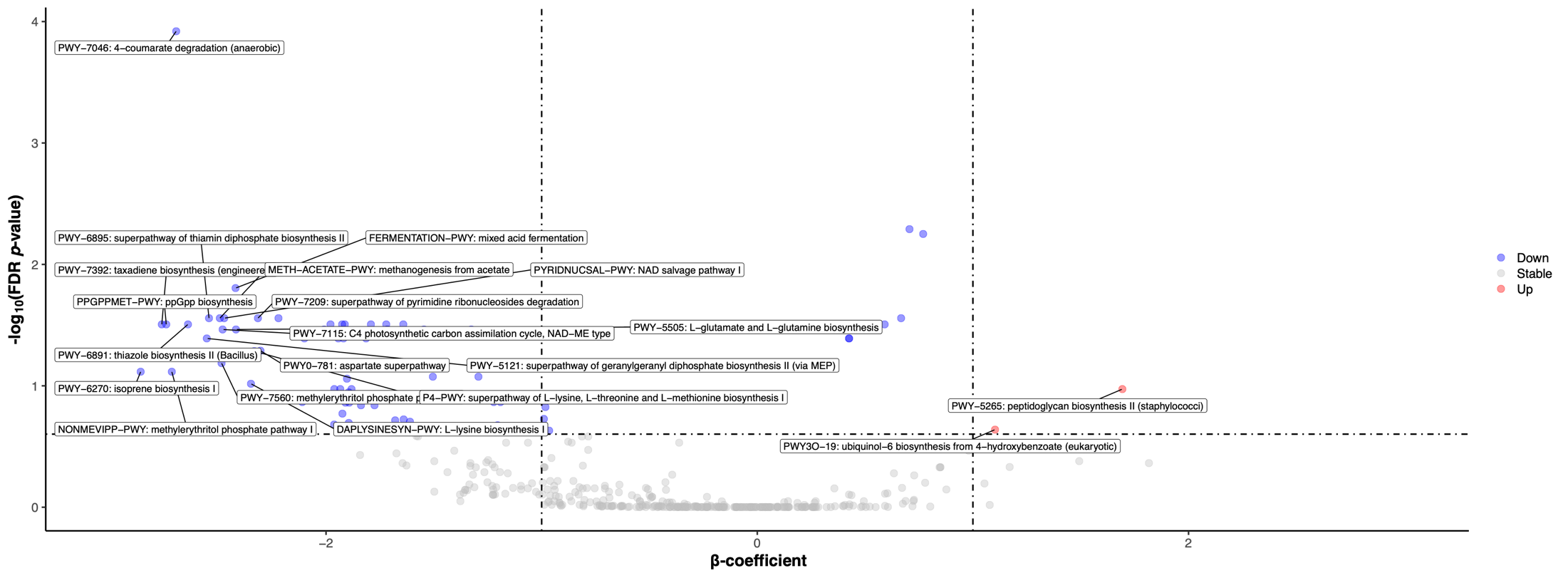
**

**Figure S4. Entropy heatmap for clinical covariates**

**
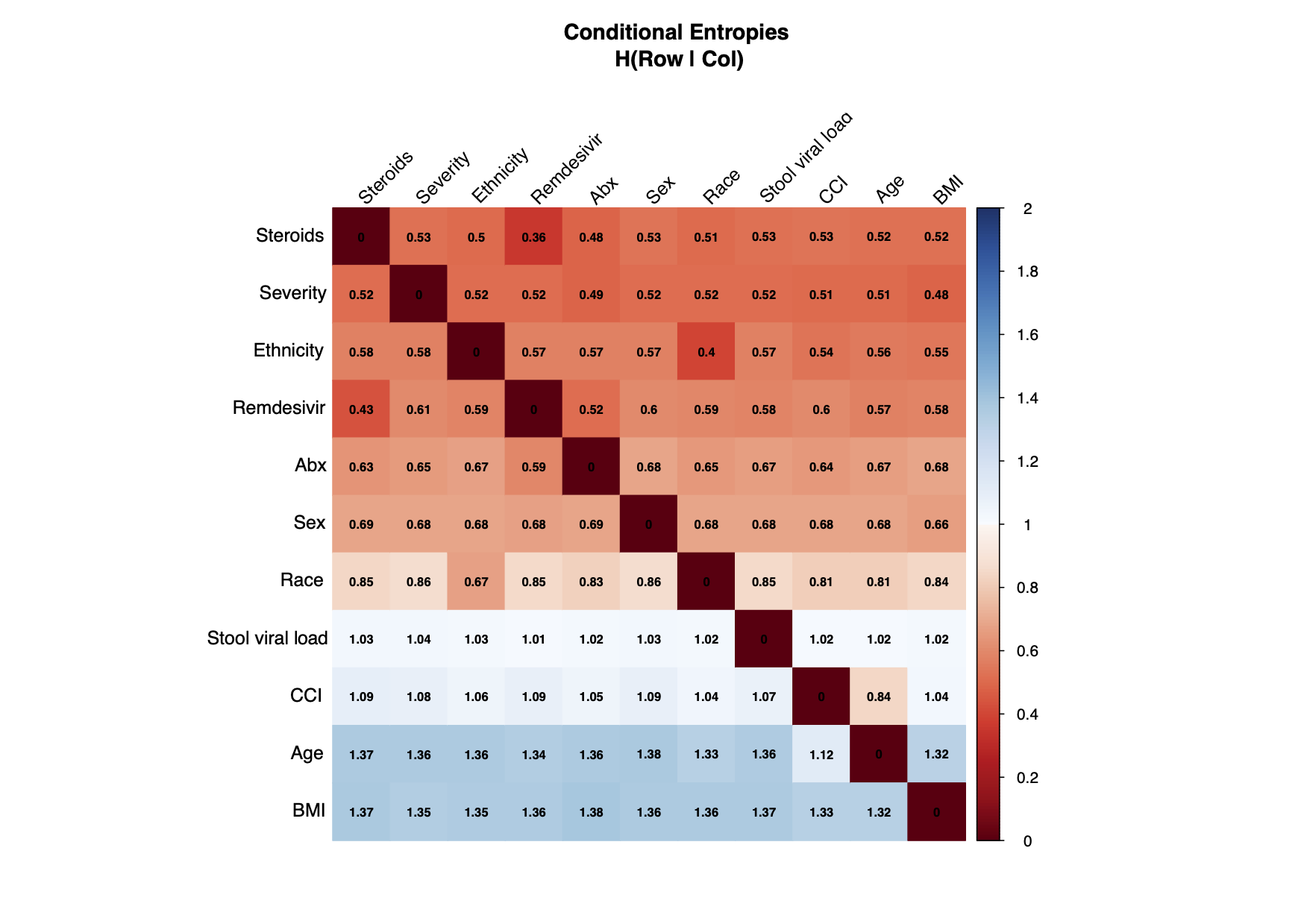
**

**Figure S5. Node map**


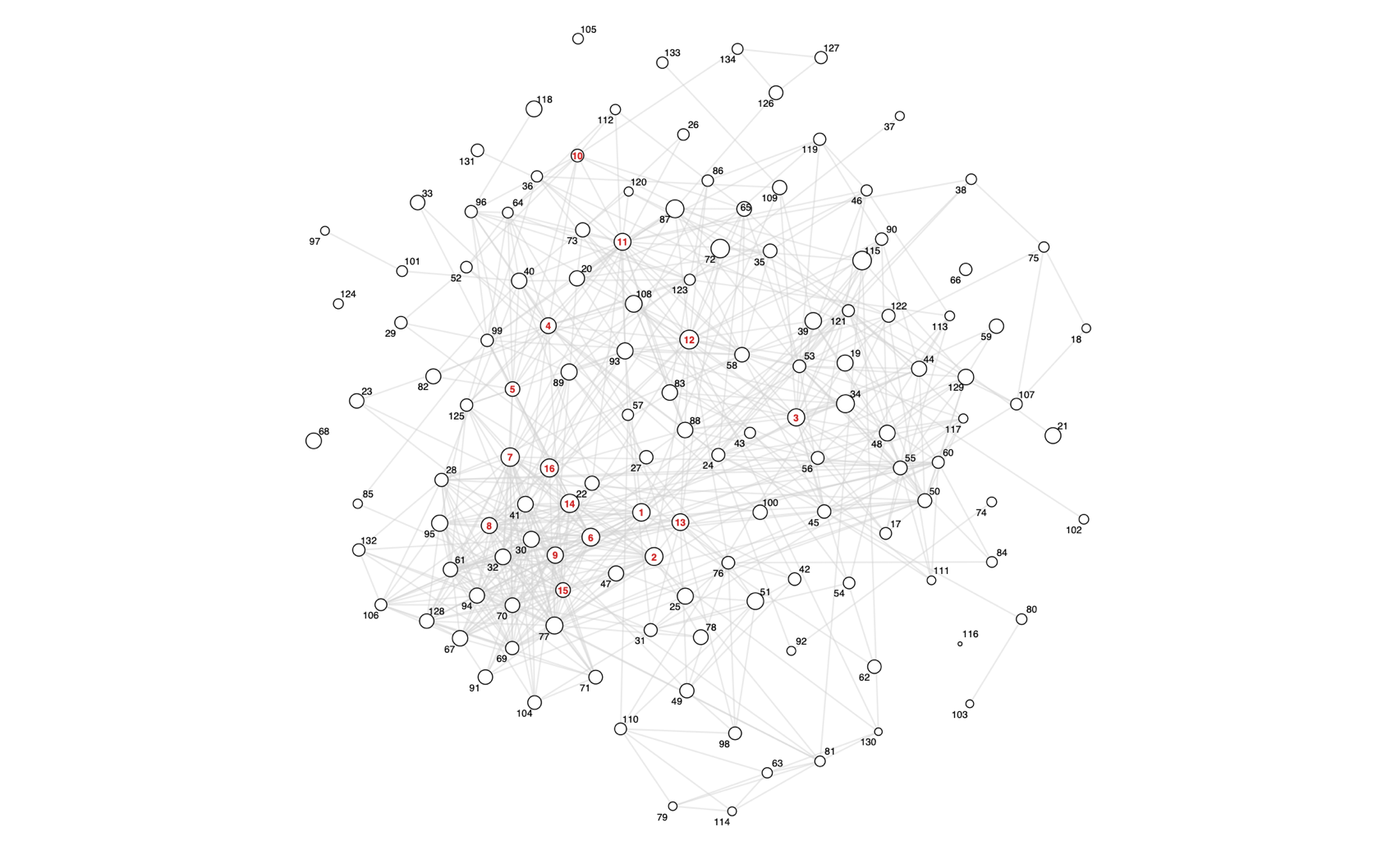


| **Number** | **Species** | **Hub Status** |
| --- | --- | --- |
| 1 | *Blautia wexlerae* | Hub |
| 2 | *Eubacterium hallii* | Hub |
| 3 | *Gordonibacter pamelaeae* | Hub |
| 4 | *Odoribacter splanchnicus* | Hub |
| 5 | *Alistipes shahii* | Hub |
| 6 | *Anaerostipes hadrus* | Hub |
| 7 | *Faecalibacterium prausnitzii* | Hub |
| 8 | *Dorea formicigenerans* | Hub |
| 9 | *Eubacterium rectale* | Hub |
| 10 | *Bacteroides massiliensis* | Hub |
| 11 | *Alistipes putredinis* | Hub |
| 12 | *Bacteroides uniformis* | Hub |
| 13 | *Streptococcus salivarius* | Hub |
| 14 | *Collinsella aerofaciens* | Hub |
| 15 | *Roseburia inulinivorans* | Hub |
| 16 | *Blautia obeum* | Hub |
| 17 | *Clostridium spiroforme* |  |
| 18 | *Enterococcus faecalis* |  |
| 19 | *Clostridium leptum* |  |
| 20 | *Parabacteroides merdae* |  |
| 21 | *Streptococcus thermophilus* |  |
| 22 | *Collinsella stercoris* |  |
| 23 | *Akkermansia muciniphila* |  |
| 24 | *Anaerotruncus colihominis* |  |
| 25 | *Bifidobacterium longum* |  |
| 26 | *Clostridium citroniae* |  |
| 27 | *Intestinimonas butyriciproducens* |  |
| 28 | *Oscillibacter* sp. CAG 241 |  |
| 29 | *Phascolarctobacterium faecium* |  |
| 30 | *Ruminococcus torques* |  |
| 31 | *Streptococcus vestibularis* |  |
| 32 | *Eubacterium eligens* |  |
| 33 | *Bacteroides dorei* |  |
| 34 | *Eggerthella lenta* |  |
| 35 | *Lawsonibacter asaccharolyticus* |  |
| 36 | *Holdemania filiformis* |  |
| 37 | *Dielma fastidiosa* |  |
| 38 | *Enterococcus faecium* |  |
| 39 | *Flavonifractor plautii* |  |
| 40 | *Methanobrevibacter smithii* |  |
| 41 | *Roseburia hominis* |  |
| 42 | *Actinomyces oris* |  |
| 43 | *Anaerofustis stercorihominis* |  |
| 44 | *Blautia hydrogenotrophica* |  |
| 45 | *Dorea sp.* CAG 317 |  |
| 46 | *Harryflintia acetispora* |  |
| 47 | *Ruminococcus bromii* |  |
| 48 | *Ruminococcus gnavus* |  |
| 49 | *Actinomyces odontolyticus* |  |
| 50 | *Blautia sp.* CAG 257 |  |
| 51 | *Streptococcus parasanguinis* |  |
| 52 | *Parabacteroides goldsteinii* |  |
| 53 | *Eisenbergiella massiliensis* |  |
| 54 | *Clostridium clostridioforme* |  |
| 55 | *Sellimonas intestinalis* |  |
| 56 | *Monoglobus pectinilyticus* |  |
| 57 | *Streptococcus anginosus group* |  |
| 58 | *Eisenbergiella tayi* |  |
| 59 | *Erysipelatoclostridium ramosum* |  |
| 60 | *Blautia producta* |  |
| 61 | *Gemmiger formicilis* |  |
| 62 | *Bacteroides fragilis* |  |
| 63 | *Lactobacillus paragasseri* |  |
| 64 | *Bacteroides salyersiae* |  |
| 65 | *Bacteroides stercoris* |  |
| 66 | *Hungatella hathewayi* |  |
| 67 | *Agathobaculum butyriciproducens* |  |
| 68 | *Escherichia coli* |  |
| 69 | *Oscillibacter sp.* 57 20 |  |
| 70 | *Coprococcus catus* |  |
| 71 | *Roseburia intestinalis* |  |
| 72 | *Bacteroides vulgatus* |  |
| 73 | *Bacteroides xylanisolvens* |  |
| 74 | *Clostridium asparagiforme* |  |
| 75 | *Lactobacillus rhamnosus* |  |
| 76 | *Streptococcus gordonii* |  |
| 77 | *Fusicatenibacter saccharivorans* |  |
| 78 | *Firmicutes bacterium* CAG 83 |  |
| 79 | *Lactobacillus gasseri* |  |
| 80 | *Bacteroides faecis* |  |
| 81 | *Lactobacillus fermentum* |  |
| 82 | *Eubacterium siraeum* |  |
| 83 | *Adlercreutzia equolifaciens* |  |
| 84 | *Anaerotignum lactatifermentans* |  |
| 85 | *Bacteroides nordii* |  |
| 86 | *Clostridium lavalense* |  |
| 87 | *Parabacteroides distasonis* |  |
| 88 | *Asaccharobacter celatus* |  |
| 89 | *Bacteroides thetaiotaomicron* |  |
| 90 | *Clostridium symbiosum* |  |
| 91 | *Bifidobacterium adolescentis* |  |
| 92 | *Ruminococcaceae bacterium* D16 |  |
| 93 | *Bacteroides ovatus* |  |
| 94 | *Coprococcus comes* |  |
| 95 | *Dorea longicatena* |  |
| 96 | *Butyricimonas virosa* |  |
| 97 | *Eubacterium callanderi* |  |
| 98 | *Rothia mucilaginosa* |  |
| 99 | *Alistipes indistinctus* |  |
| 100 | *Bilophila wadsworthia* |  |
| 101 | *Eubacterium limosum* |  |
| 102 | *Klebsiella pneumoniae* |  |
| 103 | *Bacteroides faecis* CAG 32 |  |
| 104 | *Roseburia faecis* |  |
| 105 | *Paraprevotella xylaniphila* |  |
| 106 | *Prevotella copri* |  |
| 107 | *Blautia coccoides* |  |
| 108 | *Alistipes finegoldii* |  |
| 109 | *Clostridium bolteae* |  |
| 110 | *Veillonella parvula* |  |
| 111 | *Clostridium hylemonae* |  |
| 112 | *Bacteroides finegoldii* |  |
| 113 | *Clostridium methylpentosum* |  |
| 114 | *Bifidobacterium breve* |  |
| 115 | *Ruthenibacterium lactatiformans* |  |
| 116 | *Faecalicatena orotica* |  |
| 117 | *Anaerostipes caccae* |  |
| 118 | *Bacteroides caccae* |  |
| 119 | *Bacteroides cellulosilyticus* |  |
| 120 | *Catabacter hongkongensis* |  |
| 121 | *Clostridium scindens* |  |
| 122 | *Firmicutes bacterium* CAG 145 |  |
| 123 | *Butyricimonas synergistica* |  |
| 124 | *Christensenella minuta* |  |
| 125 | *Clostridium* sp. CAG 58 |  |
| 126 | *Parasutterella excrementihominis* |  |
| 127 | *Proteobacteria bacterium* CAG 139 |  |
| 128 | *Eubacterium ramulus* |  |
| 129 | *Clostridium innocuum* |  |
| 130 | *Candida albicans* |  |
| 131 | *Barnesiella intestinihominis* |  |
| 132 | *Holdemanella biformis* |  |
| 133 | *Clostridium bolteae* CAG 59 |  |
| 134 | *Turicimonas muris* |  |
| NODE IDENTITIES MAPPED ON MODERATE NETWORK (NOTE: NODE POSITION BETWEEN MODERATE AND SEVERE NETWORKS ARE LOCKED) | | |
